# Controlling the Regioselectivity of Topochemical Reduction Reactions Through Sequential Anion Insertion and Extraction

**DOI:** 10.1002/anie.202514045

**Published:** 2025-09-09

**Authors:** Romain Wernert, Bodoo Batnaran, Michael A. Hayward

**Affiliations:** ^1^ Department of Chemistry Inorganic Chemistry Laboratory University of Oxford South Parks Road Oxford OX1 3QR UK

**Keywords:** Mixed‐anion compounds, Regioselectivity control, Ruddlesden‐Popper oxyfluorides, Topochemical reactions, Transition‐metal oxides

## Abstract

Topochemical reduction of the *n* = 2 Ruddlesden‐Popper oxide, LaSr_2_CoRuO_7_, yields LaSr_2_CoRuO_5.3_, a phase containing (Co/Ru)O_4_ squares which share corners to form 1D infinite double‐chains. In contrast, fluorination of LaSr_2_CoRuO_7_ yields the oxyfluoride LaSr_2_CoRuO_5.5_F_3.5_, which can then be reduced to form LaSr_2_CoRuO_4.5_F_1.5_. This reduced oxyfluoride is almost isoelectronic with LaSr_2_CoRuO_5.3_, but LaSr_2_CoRuO_4.5_F_1.5_ has a crystal structure in which the (Co/Ru)O_4_ squares are connected into 2D infinite sheets. Thus, by following a fluorinate‐then‐reduce reaction scheme, the regiochemistry of topochemical reduction reactions can be modified, and compounds with different transition‐metal‐centre interconnectivity can be prepared. Both LaSr_2_CoRuO_5.3_ and LaSr_2_CoRuO_4.5_F_1.5_ adopt glassy magnetic states at low temperature, but the magnetic interactions present in LaSr_2_CoRuO_5.3_ appear to be significantly stronger than those in LaSr_2_CoRuO_4.5_F_1.5_, attributable to the differing dimensionality of the transition‐metal connectivity. The structural features of LaSr_2_CoRuO_5.5_F_3.5_ that modify the regioselectivity of the topochemical reduction reaction appear to be common to many fluorinated Ruddlesden‐Popper oxides, suggesting this fluorinate‐then‐reduce strategy could be used to prepare a range of “infinite‐layer” reduced phases which cannot be made by direct reduction of Ruddlesden‐Popper oxide precursors.

## Graphical abstract

Topochemical reduction reactions allow the preparation of metastable materials containing low‐valent transition‐metal cations. By adopting a fluorinate‐then‐reduce reaction scheme, the regioselectivity of these reactions can be modified, allowing the connectivity of the transition‐metal coordination polyhedra in the resulting materials (1D vs. 2D) to be controlled.

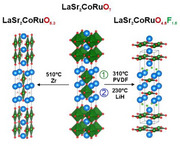

Complex transition‐metal oxides exhibit a wide range of physical properties, from superconductivity, magnetoresistance and magnetoelectricity to a diverse variety of complex magnetic behaviour.^[^ ^1^, ^2^, ^3^, ^4^ ^]^ These properties arise from the presence of electrons in partially occupied transition‐metal *d*‐states and their subsequent intra‐ and inter‐atomic interactions.^[^ ^3^, ^4^ ^]^ With this in mind, one approach to tuning the physical behaviour of transition‐metal oxide systems is to make changes to factors that determine the local electronic states of the transition‐metal centres (oxidation state, local coordination environment) and to also modify the coupling between these states by changing manner in which the coordinated metal centres are physically connected to each other within the crystal lattice of materials.

Typically, these changes are enacted by modifying the cation lattice of materials via substitution of the transition‐metal cations and/or substitution, insertion or removal of charge‐balancing “spectator” cations.^[^ ^5^ ^]^ However, modification of the anion lattice can also be utilised to achieve the same goal.^[^ ^6^ ^]^ For example, partial fluoride‐for‐oxide substitution in the LaFeAsO_1‐_ *_x_* F*_x_* system can convert the undoped antiferromagnetic semiconductor (*x* = 0) into a metallic phase which superconducts below *T*_c_ = 26 K (*x* = 0.11).^[^ ^7^ ^]^

While “anion‐doping” is a powerful approach to tuning the behaviour of transition‐metal oxide systems, in some cases it is hard to realise in practice because the desired mixed‐anion compound is metastable with respect to the all‐oxide alternative. This issue can be partially overcome by using post‐synthesis, topochemical reactions to modify the anion framework of a parent phase via anion‐insertion, ‐extraction or ‐exchange reactions, as this class of reaction allows the preparation of metastable phases. Thus, for example, the Cr(IV) phase, La_2_SrCr_2_O_7_F_2_, can be prepared via the low‐temperature fluorination of La_2_SrCr_2_O_7_,^[^ ^8^ ^]^ or the V(III) oxyhydride phase SrVO_2_H can be prepared via topochemical anion exchange from the V(IV) perovskite oxide SrVO_3_.^[^ ^9^ ^]^

In this latter example, the substitution of O^2–^ oxide ions with H^–^ hydride ions not only leads to a reduction in the vanadium oxidation state but also changes the local symmetry at vanadium and thus the energy ordering of the vanadium *d*‐orbitals due to the ordering of the anions to form *trans*‐VO_4_H_2_ units. Furthermore, this ordering of the oxide and hydride ions within the anion framework changes the “orbital connectivity” of the material, as the lack of anion *p*‐orbitals prevents V‐H‐V linkages percolating π‐symmetry bands.^[^ ^10^ ^]^

These examples demonstrate that “anion doping” can both change the local electronic state of transition‐metal cations and tune how these local states interact. However, if we wish to make directed changes to the electronic structure of materials via anion doping, we need to be able to control the anion ordering or anion‐vacancy ordering induced via topochemical reactions.

Many topochemical reactions show regioselectivity trends. For example, when anion exchange or anion deintercalation reactions are applied to layered Ruddlesden‐Popper oxide phases, they are observed to preferentially substitute or remove the anions from “equatorial” sites (those which link neighbouring MO_6_ units) rather than from “apical” sites (those which project into the rock salt layers of the framework) in the majority of cases.^[^ ^11^ ^]^ Thus, the topochemical hydride‐for‐oxide anion exchange of the *n* = 1 Ruddlesden‐Popper oxide LaSr_3_NiRuO_8_ yields LaSr_3_NiRuO_4_H_4_,^[^ ^12^ ^]^ a phase in which all of the equatorial oxide ions have been replaced by hydride. Similarly, the reductive topochemical anion‐extraction of LaSr_3_NiRuO_8_ yields LaSr_3_NiRuO_6_, in which the induced anion vacancies are located in equatorial anion sites, resulting in an array of corner‐linked Ni^1+^O_4_ and Ru^2+^O_4_ square‐planar units which form 1D chains through the reduced material.^[^ ^13^ ^]^

Here we describe the use of back‐to‐back sequential topochemical reactions to modify the regioselectivity trends observed during the topochemical reduction of Ruddlesden‐Popper oxides to control the transition‐metal connectivity within materials.

The *n* = 2 Ruddlesden‐Popper oxide LaSr_2_CoRuO_7_ was fluorinated by the decomposition of polyvinylidene difluoride (PVDF) at 300 °C, as described in the Supporting Information. The characterisation of oxyfluoride phases is complicated by poor scattering contrast between oxide and fluoride ions in both X‐ray and neutron diffraction measurements. We therefore adopted a strategy of first determining the crystal structure of oxyfluoride phases by synchrotron X‐ray diffraction (SXRD) and neutron powder diffraction (NPD), without differentiating between the two anions. This allows us to determine the total anion content of the phase. We then used a combination of spectroscopy and titrations to determine the transition‐metal oxidation states to determine the O/F ratio. Finally, we use bond valence sums (BVS) to determine the oxide/fluoride distribution across the different anion sites. Thus, SXRD and NPD data from fluorinated LaSr_2_CoRuO_7_ were indexed with a monoclinic unit cell with reflection conditions consistent with the *A*2/*a* (#15) space group (Figures S2 and S3). A model based on the reported crystal structure of La_2_SrCr_2_O_7_F_2_^[^ ^8^ ^]^ was constructed and refined simultaneously against the SXRD and NPD data and converged readily to yield a structure with a dramatically expanded *c* lattice parameter (*c* = 23.43 Å) compared to LaSr_2_CoRuO_7_ (*c* = 20.36 Å) and additional anions located in tetrahedral interlayer interstitial sites, as shown in Figure 1, to give a compound of overall composition of LaSr_2_CoRu(O/F)_9_. Iodometric titrations confirm the total transition‐metal charge as +7.5, consistent with the Ru^5+^, Co^2+/3+^ combination determined from Ru and Co K‐edge XANES data (Figure 2) and thus an overall composition of LaSr_2_CoRuO_5.5_F_3.5_ for the phase. BVS values, described in detail in the Supporting Information (Table S5), indicate fluoride ions occupy the tetrahedral interstitial sites and partially occupy the apical anion sites, as shown in Figure 1, in a similar way as observed for La_3_Ni_2_O_5.5_F_3.5_ and Sr_3_FeRuO_5.5_F_3.5_.^[^ ^14^, ^15^ ^]^

**Figure 1:**
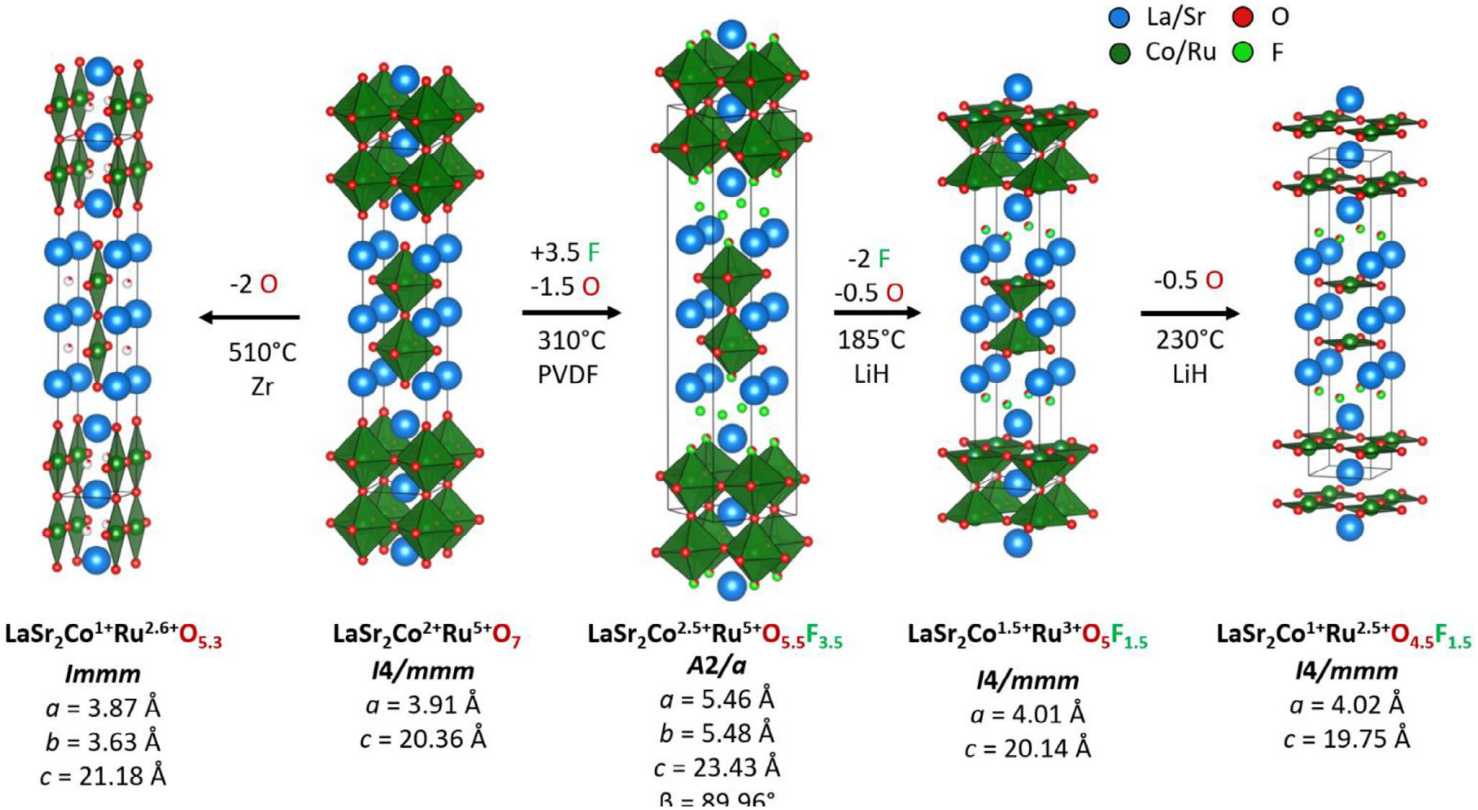
Summary of topochemical reaction scheme.

**Figure 2:**
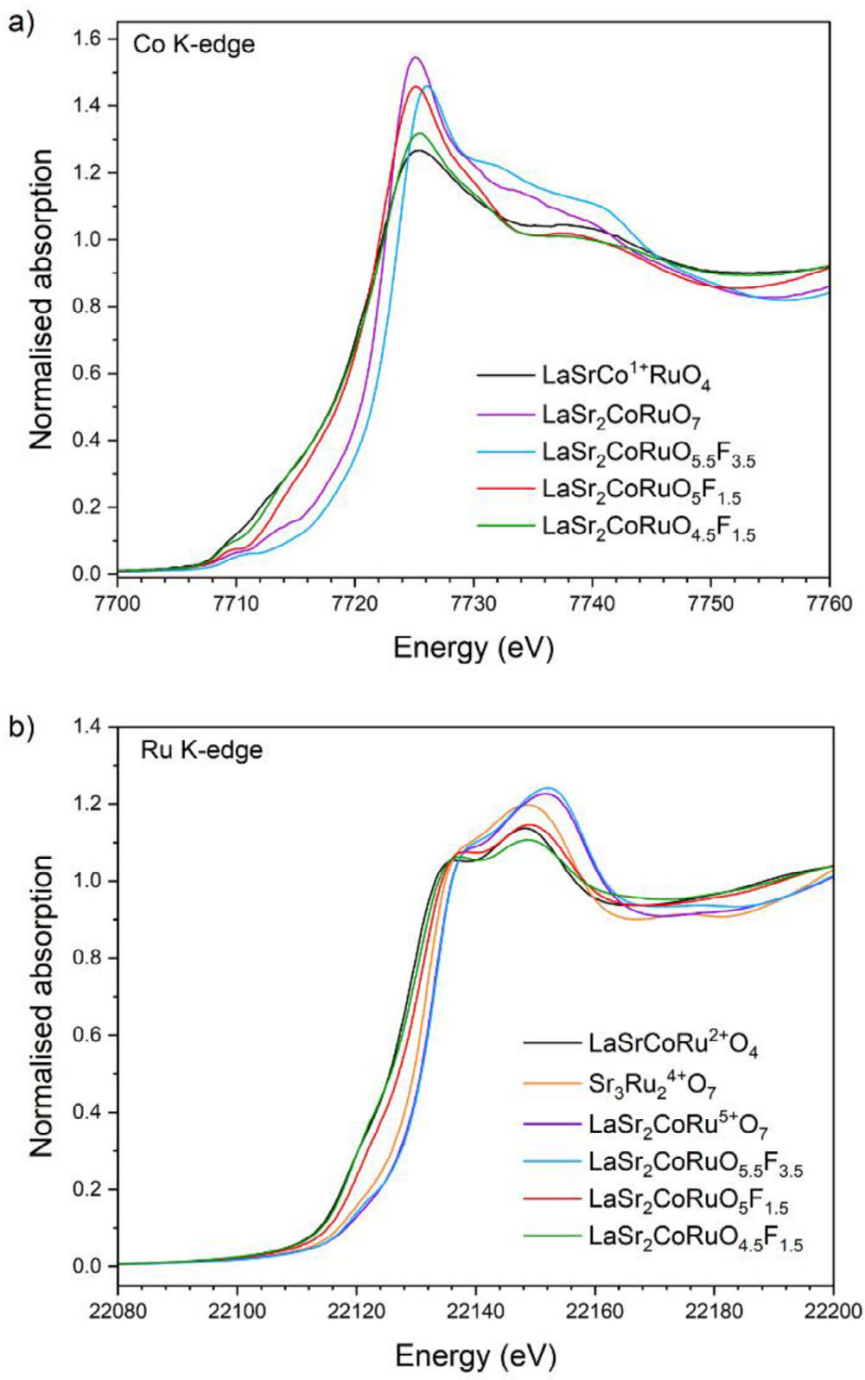
X‐ray absorption near‐edge spectra collected at Co and Ru K‐edges for LaSr_2_CoRuO_7_, LaSr_2_CoRuO_5.5_F_3.5_, LaSr_2_CoRuO_5_F_1.5_ and LaSr_2_CoRuO_4.5_F_1.5_. The spectra are referenced against LaSrCo^+^Ru^2+^O_4_ and Sr_3_Ru_2_^4+^O_7_.

Reaction of LaSr_2_CoRuO_5.5_F_3.5_ with LiH at 185 °C yields LaSr_2_CoRuO_5_F_1.5_, which is converted to LaSr_2_CoRuO_4.5_F_1.5_ on further reaction at 230 °C. NPD data (Figures S8, S9, S12 and S13) indicate that the tetrahedral interstitial anion sites remain fully occupied in both reduced phases, with anions only removed from the apical and bridging anion sites on reduction, as shown in Figure 1 and in detail in the Supporting Information. Again, the anion composition and distribution of the reduced phases were deduced from a combination of TGA and XANES data and BVS calculations (Figures S11, S12, S16 and S17).

Thus, the sequential fluorination and then reduction of LaSr_2_CoRuO_7_ leads to a phase, LaSr_2_CoRuO_4.5_F_1.5_ with the T’ crystal structure analogous to La_3_Ni_2_O_6_,^[^ ^16^ ^]^ containing 2D infinite sheets of (Co/Ru)O_4_ squares. In contrast, direct reduction of LaSr_2_CoRuO_7_ without prior fluorination yields LaSr_2_CoRuO_5.3_ with a structure consisting of 1D double chains of (Co/Ru)O_4_ squares, as shown in Figure 1 and detailed in the Supporting Information, highlighting the reaction‐path dependence of the product phases formed.

This striking difference between the product of the fluorinate‐then‐reduce procedure and that of direct reduction indicates that the insertion/exchange of fluoride ions into the LaSr_2_CoRuO_7_ framework changes the regioselectivity of the subsequent reduction reaction, favouring the removal of apical and bridging anions from LaSr_2_CoRuO_5.5_F_3.5_, in contrast to the observed removal of anions from equatorial anion sites during the reduction of LaSr_2_CoRuO_7_.

As noted above, the preference to remove equatorial anions during the topochemical reactions of *n* = 1 and *n* = 2, A*_n_*_+1_B*_n_* O_3_ *_n_*_+1_ Ruddlesden‐Popper oxides is widely observed.^[^ ^11^, ^13^, ^17^, ^18^, ^19^, ^20^ ^]^ This regioselectivity can be rationalised by noting that the anions removed tend to have stronger bonding interactions (as evaluated by bond valence sums^[^ ^21^ ^]^) with the redox‐active B‐cations than the redox‐inactive A‐cations, so removal or substitution of these anions results in product phases with lower lattice strain, as discussed previously.^[^ ^11^ ^]^ This “bond valence sum strain” (BVSS) analysis accounts for the regioselectivity of the direct reduction of LaSr_2_CoRuO_7_, but not that of LaSr_2_CoRuO_5.5_F_3.5_, where the BVSS analysis predicts the removal of equatorial anions, as described in Tables S9–S12.

Structural analysis of the fluorinated phases reveals that insertion and exchange of fluoride ions into the interstitial and apical anion sites, respectively, of LaSr_2_CoRuO_7_, perturbs the bonding in the system, principally by introducing a series of short anion‐anion contacts between the apical and interstitial anion sites (<(O/F)_ap_–F_int_> = 2.77 Å), neighbouring pairs of interstitial sites (<F_int_–F_int_> = 2.74 Å>) and the apical and equatorial anion sites (<(O/F)_ap_–O_eq_> = 2.72 Å), which is accompanied by a contraction of the (Co/Ru)–(O/F)_ap_ bond (1.910(24) Å) and an expansion of the (Co/Ru)–O_bridging_ bond (1.997(5) Å), as shown in Figure 3.

**Figure 3:**
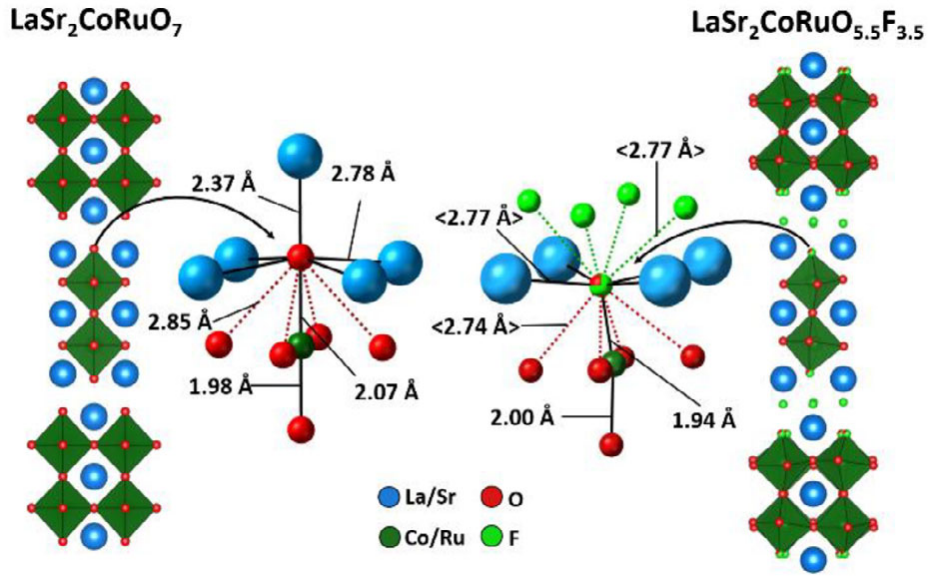
Local structure around apical anion site in LaSr_2_CoRuO_7_ (left) and LaSr_2_CoRuO_5.5_F_3.5_ (right).

Removal of the apical anions during the formation of LaSr_2_CoRuO_5_F_1.5_ relieves this anion‐anion repulsion and also allows the coordination sphere of the rock salt layer A‐cation to contract from a large 12‐coordinate site to a small 8‐coordinate site, with a concomitant increase in bond valence sum (Sr + 2.20 increasing to Sr + 2.42). These changes appear to override the BVSS regioselectivity trends, leading to a phase, LaSr_2_CoRuO_5_F_1.5_, with 2D connectivity, rather than the 1D connectivity of a LaSr_2_CoRuO_5_–like phase.

In principle the reduction of LaSr_2_CoRuO_5_F_3.5_ could proceed via the removal of the interstitial anions, which would also lower the F_int_–(O/F)_ap_ anion repulsion. However, this deintercalation would form ‘LaSr_2_CoRuO_5.5_F_1.5′_ in which the rock salt A‐cations would reside in 9‐coordinate sites with an average composition of O_5.25_F_3.75_, which would leave the La^3+^ and Sr^2+^ cations significantly under bonded in the absence of a large structural distortion to shorten the (La/Sr)─(O/F) bond lengths.

Figure 3 shows that in addition to introducing short F_int_–(O/F)_ap_ contacts, the insertion of fluoride into interlayer interstitial sites compresses the perovskite blocks, shortening the (Co/Ru)─(O/F)_ap_ bond and the (O/F)_ap_–O_eq_ separation. This compression appears to be a common feature of topochemically fluorinated *n* = 2 Ruddlesden‐Popper phases,^[^ ^8^, ^14^, ^15^, ^22^, ^23^, ^24^, ^25^ ^]^ suggesting the changes in reduction‐reaction regioselectivity observed in fluorinated LaSr_2_CoRuO_7_ will be common to other fluorinated *n* = 2 Ruddlesden‐Popper oxides, and so this strategy could be used to prepare a range of ‘infinite‐layer’ reduced phases which cannot be made by direct reduction of *n* = 2 Ruddlesden‐Popper precursors.

Magnetisation data, described in detail in Figures S21–S30, indicate LaSr_2_CoRuO_7_ adopts a canted antiferromagnetic state below *T*_N_ ∼ 50 K. However, the chemical disorder introduced on fluorination to form LaSr_2_CoRuO_5.5_F_3.5_ appears to frustrate the magnetic couplings in the oxyfluoride phase, leading to a glassy magnetic state below *T* ∼ 50 K. The topochemically reduced phases LaSr_2_CoRuO_5_F_1.5_, LaSr_2_CoRuO_4.5_F_1.5_ and LaSr_2_CoRuO_5.3_ also adopt glassy states at low temperature, so it is hard to establish definitive structure‐property relations for these compounds. However, it is interesting to note that, while the Curie constants extracted for LaSr_2_CoRuO_4.5_F_1.5_ and LaSr_2_CoRuO_5.3_ are similar, consistent with their similar transition‐metal oxidation states, LaSr_2_CoRuO_5.3_ exhibits a larger Weiss constant (*θ* = −147.3 K) and deviates from the Curie‐Weiss law at a higher temperature (*T*_dev_ ∼ 175 K) than LaSr_2_CoRuO_4.5_F_3.5_ (θ = ‐48.3 K; *T*_dev_ ∼ 95 K) suggesting that the connectivity of the (Co/Ru)O_4_ units in 1D LaSr_2_CoRuO_5.3_ versus 2D LaSr_2_CoRuO_4.5_F_1.5_ does have a strong influence on the strength of the magnetic couplings in these systems.

## Supporting Information

The authors have cited additional references within the Supporting Information.^[^ ^26^ ^]^

## Conflict of Interests

The authors declare no conflict of interest.

## Supporting information



Supporting Information

## Data Availability

The data that support the findings of this study are openly available in STFC data archive at doi.org/10.5286/ISIS.E.RB2420082‐1.
