# Supplementary material for: Controlling the Regioselectivity of Topochemical Reduction Reactions Through Sequential Anion Insertion and Extraction
Source: Angew Chem Int Ed Engl. 2025 Sep 9;64(43):e202514045. doi: 10.1002/anie.202514045 (PMC12535380; doi:10.1002/anie.202514045)
Supplement: Supplementary file 1 — Supporting Information [file ANIE-64-e202514045-s001.pdf]

Controlling the Regioselectivity of Topochemical Reduction Reactions through Sequential Anion Insertion and Extraction

Romain Wernert, Bodoo Batnaran and Michael A. Hayward*

Supporting Information

Table of Contents

1. Sample Preparation
Synthesis of $LaSr_2CoRuO_7$.
Figure S1. Observed calculated and difference plots from the structural refinement of $LaSr_2CoRuO_7$ against SXRD data.
Table S1. Crystallographic parameters extracted from the structural refinement of $LaSr_2CoRuO_7$ against SXRD data.
Fluorination of $LaSr_2CoRuO_7$.
Topochemical reduction.

2. Characterization methods

3. Characterization of $LaSr_2CoRuO_{5.5}F_{3.5}$
Figure S2. Observed, calculated and difference plots from the structural refinement of an *A2/a* symmetry model against SXRD data collected from $LaSr_2CoRuO_{5.5}F_{3.5}$ at room temperature.
Figure S3. Observed, calculated and difference plots from the structural refinement of an *A2/a* symmetry model of $LaSr_2CoRuO_{5.5}F_{3.5}$ against NPD data collected using the 5 detector banks of the POLARIS instrument at room temperature.
Table S2. Parameters extracted from the structural refinement of $LaSr_2CoRuO_{5.5}F_{3.5}$ against SXRD and NPD data collected at room temperature.
Table S3. Bond valence sums of the different anion sites in $LaSr_2CoRuO_{5.5}F_{3.5}$ calculated for either a full occupancy by F^- or O^2.
Figure S4. Crystal structure and labelling of anion sites in $LaSr_2CoRuO_{5.5}F_{3.5}$
Figure S5. X-ray absorption near edge spectra (XANES) collected at Co and Ru K-edges for $LaSr_2CoRuO_7$ and $LaSr_2CoRuO_{5.5}F_{3.5}$.
Figure S6. Thermogravimetric analysis data collected from $LaSr_2CoRuO_{5.5}F_{3.5}$ while heated under a flowing 10% H_2, 90% N_2 atmosphere.
Figure S7. X-ray powder diffraction data collected from the sample after thermogravimetric analysis under a flowing 10% H_2, 90% N_2 atmosphere.

4. Characterization of $LaSr_2CoRuO_5F_{1.5}$
Figure S8. Observed, calculated and difference plots from the structural refinement of an *I4/mmm* symmetry model against SXRD data collected from $LaSr_2CoRuO_5F_{1.5}$ at room temperature.
Figure S9. Observed, calculated and difference plots from the structural refinement of an *I4/mmm* symmetry model against NPD data collected from $LaSr_2CoRuO_5F_{1.5}$ using the 5 detector banks of the POLARIS instrument at room temperature.
Table S4. Parameters extracted from the structural refinement of $LaSr_2CoRuO_5F_{1.5}$ against SXRD and NPD data collected at room temperature.
Table S5. Bond valence sums of the different anion sites in $LaSr_2CoRuO_5F_{1.5}$ calculated for either a full occupancy by F^- or O^2.
Figure S10. Crystal structure and labelling of anion sites in $LaSr_2CoRuO_5F_{1.5}$.
Figure S11. X-ray absorption near edge spectra collected at Co and Ru K-edges from $LaSr_2CoRuO_5F_{1.5}$.
Figure S12. Thermogravimetric analysis curves for $LaSr_2CoRuO_5F_{1.5}$ under flowing 10% H_2/N_2 gas.

5. Characterization of $LaSr_2CoRuO_{4.5}F_{1.5}$
Figure S13. Observed, calculated and difference plots from the structural refinement of an *I4/mmm* symmetry model against SXRD data collected from $LaSr_2CoRuO_{4.5}F_{1.5}$ at room temperature.
Figure S14. Observed, calculated and difference plots from the structural refinement of an *I4/mmm* symmetry model against NPD data collected from $LaSr_2CoRuO_{4.5}F_{1.5}$ using the 5 detector banks of the POLARIS instrument at room temperature.
Table S6. Parameters extracted from the structural refinement of $LaSr_2CoRuO_{4.5}F_{1.5}$ against SXRD and NPD data collected at room temperature.

Table S7. Bond valence sums of the different anion sites in $LaSr_2CoRuO_{4.5}F_{1.5}$ calculated for either a full occupancy by F^- or O^2.
Figure S15. Crystal structure and labelling of anion sites in $LaSr_2CoRuO_{4.5}F_{1.5}$
Figure S16. X-ray absorption near edge spectra collected at Co and Ru K-edges for $LaSr_2CoRuO_{4.5}F_{1.5}$.
Figure S17. Thermogravimetric analysis curves for $LaSr_2CoRuO_{4.5}F_{1.5}$ under flowing 10% H_2/N_2 gas.

6 Characterization of $LaSr_2CoRuO_{5+\delta}$
Figure S18. Observed, calculated and difference plots from the structural refinement of an *Immm* symmetry model against SXRD data collected from $LaSr_2CoRuO_{5.3}$ at room temperature.
Table S8. Crystallographic parameters from structural refinement of $LaSr_2CoRuO_{5.3}$ against SXRD data.
Figure S19. Thermogravimetric analysis curves for $LaSr_2CoRuO_5$ under flowing O_2 gas.

7. Normalized Co and Ru K-edge XANES spectra
Figure S20. Normalized X-ray absorption spectra at Co and Ru K-edges.

8. Bond Valence Sum Strain (BVSS) calculations for $LaSr_2CoRuO_xF_y$ phases.
Table S9. Calculated bond valence sums for the three anion sites in $LaSr_2CoRuO_7$ and their separated contributions from the A-cations (Co/Ru) and B-cations (La/Sr) in the structure.
Table S10. Calculated bond valence sums for the three anion sites in $LaSr_2CoRuO_{5.5}F_{3.5}$ and their separated contributions from the A-cations (Co/Ru) and B-cations (La/Sr) in the structure.
Table S11. Calculated anion bond valence sums for a series of $A_3B_2O_7$ n = 2 Ruddlesden-Popper oxides and their fluorinated products.
Table S12. Calculated bond valence sums for the three anion sites in $LaSr_2CoRuO_5F_{1.5}$ and their separated contributions from the A-cations (Co/Ru) and B-cations (La/Sr) in the structure.

9. Magnetic characterization
Magnetic characterization of $LaSr_2CoRuO_7$
Figure S21. Zero-field cooled and field-cooled magnetization data collected from $LaSr_2CoRuO_7$ as a function of temperature in an applied field of 100 Oe.
Figure S22. Plot of reciprocal of ZFC magnetization data collected from $LaSr_2CoRuO_7$ as a function of temperature in an applied field of 100 Oe.
Figure S23. Magnetization data collected from $LaSr_2CoRuO_7$ at 300 K and 5 K, as a function of applied field.
Magnetic characterization of $LaSr_2CoRuO_{5.5}F_{3.5}$
Figure S24. Zero-field cooled and field-cooled magnetization data collected from $LaSr_2CoRuO_{5.5}F_{3.5}$ as a function of temperature in an applied field of 100 Oe.
Figure S25. Plot of reciprocal of ZFC magnetization data collected from $LaSr_2CoRuO_{5.5}F_{3.5}$ as a function of temperature in an applied field of 100 Oe.
Figure S26. Magnetization data collected from $LaSr_2CoRuO_{5.5}F_{3.5}$ at 300 K and 5 K, as a function of applied field.
Magnetic characterization of $LaSr_2CoRuO_5F_{1.5}$, $LaSr_2CoRuO_{4.5}F_{1.5}$ and $LaSr_2CoRuO_{5.3}$
Figure S27. Magnetization-field data collected at 300 K from $LaSr_2CoRuO_5F_{1.5}$, $LaSr_2CoRuO_{4.5}F_{1.5}$ and $LaSr_2CoRuO_{5.3}$.
Figure S28. Plots of paramagnetic susceptibility and saturated ferromagnetic moment, as a function of temperature, for $LaSr_2CoRuO_5F_{1.5}$, $LaSr_2CoRuO_{4.5}F_{1.5}$ and LaSr $CoRuO_{5.3}$ as determined by the 'ferrosubtraction method'.
Figure S29. Plots of the inverse paramagnetic susceptibilities of $LaSr_2CoRuO_5F_{1.5}$, $LaSr_2CoRuO_{4.5}F_{1.5}$ and $LaSr_2CoRuO_{5.3}$ against temperature.
Figure S30. Magnetization-field data collected at 5 K after cooling in 50000 Oe from 300 K, from $LaSr_2CoRuO_5F_{1.5}$, $LaSr_2CoRuO_{4.5}F_{1.5}$ and $LaSr_2CoRuO_{5.3}$.

10. Magnetic measurements in the presence of elemental Co impurities via the 'ferrosubtraction' method
Figure S31. Magnetization of $LaSr_2CoRuO_{4.5}F_{1.5}$ measured as a function of applied field at 300 K.

11. References

1. Sample Preparation

Synthesis of $LaSr_2CoRuO_7$. 3 gram samples of $LaSr_2CoRuO_7$ were prepared by a high temperature ceramic synthesis method. Suitable stoichiometric ratios of Co_3O_4 (99.99%), $SrCO_3$ (99.99%), RuO_2 (99.99%, dried for 3 hours at 800°C) and La_2O_3 (99.99%, dried overnight at 900 °C) were mixed together and ground in an agate pestle and mortar. The mixture was first heated in air at 900 °C for 12 h, ground again and then pressed into pellets prior to being heated to 1300 °C for multiple 48 h periods until no further change was observed in powder X-ray diffraction data. Diffraction data collected from the final product could be readily indexed with a tetragonal unit cell with systematic absences consistent with space group *I*4/*mmm* and was fit by a model based on the reported structure of $LaSr_2NiRuO_7$.[1]

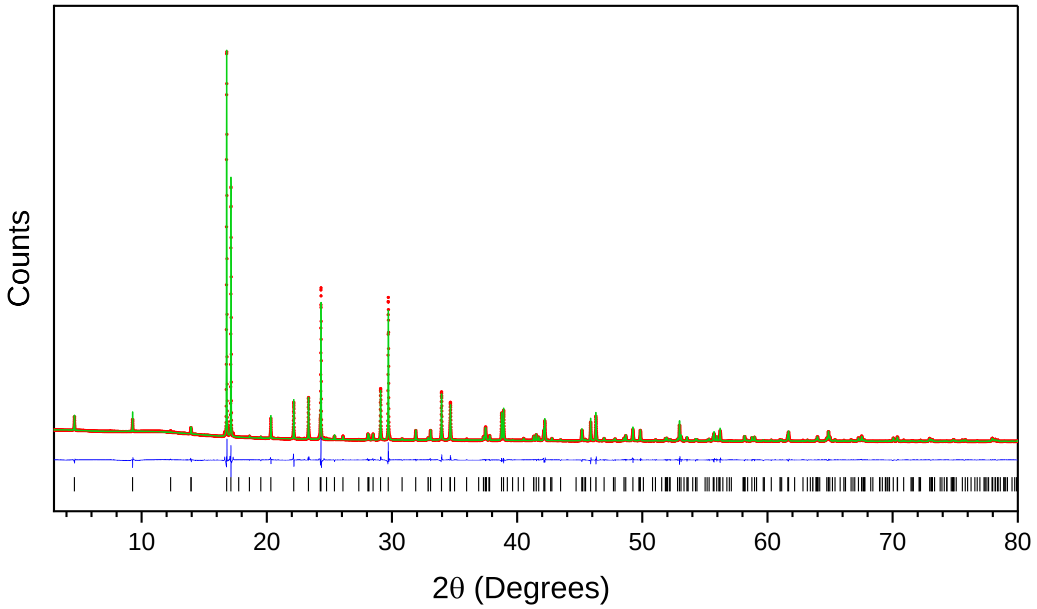

Figure S1. Observed calculated and difference plots from the structural refinement of $LaSr_2CoRuO_7$ against SXRD data.

Atom	site	*x*	*y*	*z*	Occupancy	B_{iso} (Å^2)
La/Sr(1)	2*b*	0	0	½	0.333 / 0.666	0.68(2)
La/Sr(2)	4*e*	0	0	0.31594(3)	0.333 / 0.666	0.55(2)
Co/Ru	4*e*	0	0	0.09723(2)	0.5 / 0.5	0.19(2)
O(1)	2*a*	0	0	0	1	1.06(4)
O(2)	8*g*	0	½	0.0968(2)	1	1.06(4)
O(3)	4*e*	0	0	0.1992(2)	1	1.06(4)
	$LaSr_2CoRuO_7$, space group *I*4/*mmm* (# 139)<br>*a* = 3.90675(2) Å, *c* =20.3569(7) Å, V = 310.701(2) Å^3<br>Formula weight = 586.142 g·mol^{-1}, Z = 2					
	Radiation source: Synchrotron X-ray radiation (λ = 0.824 Å)<br>Temperature: 300 K<br>R_{wp} = 5.45%, R_p = 3.31%, R_{exp} = 2.63%					

Table S1. Crystallographic parameters extracted from the structural refinement of $LaSr_2CoRuO_7$ against SXRD data.

Fluorination of $LaSr_2CoRuO_7$. 1 g of $LaSr_2CoRuO_7$ was mixed with 200 mg of polyvinylidene difluoride powder (PVDF, Fluorochem) using a pestle and mortar until homogenous. The mixture was poured in an alumina crucible, and heated under flowing oxygen at 310 °C for 8 hours. This treatment was repeated twice (with addition of the same amount PVDF) after which no further change was observed in the powder X-ray diffraction data collected from the product.

Topochemical reduction. Samples of fluorinated $LaSr_2CoRuO_7$ were reduced by reaction with LiH. Test reactions to assess reactivity were performed on small samples (~250 mg) which were ground together with 6 mol equivalents of LiH in an argon-filled glovebox. The mixtures were sealed within evacuated borosilicate glass tubes and heated for periods of 2 days at temperatures ranging between 150 °C (no reaction) and 250 °C (decomposition). Larger samples (~1.25 g) used for subsequent analysis were prepared as described above using larger ampoules to minimize H_2 pressure build up. Following the reaction, LiH-reduced samples were washed with methanol to remove unreacted LiH and the Li_2O by-product before being dried under vacuum.

$LaSr_2CoRuO_7$ was also reduced using a Zr metal getter. A sample of Lar_2CoRuO_7 was sealed in a silica ampoule with powdered Zr such that the two powders share an atmosphere but were not in physical contact. The reaction apparatus was then heated at 510 °C for 3 periods of 48 h. Reduced samples were kept in an Ar-filled glovebox and handled under inert conditions.

2. Characterization methods

Reaction progress was monitored and initial structural characterizations were performed using X-ray powder diffraction data collected using a Bruker D8 Advance diffractometer equipped with a LYNXEYE silicon strip detector and operating with Cu K_{α} radiation. Air sensitive samples were measured in airtight sample holders under argon.

High resolution synchrotron powder X-ray diffraction (SXRD) data were collected using the I11 instrument at Diamond Light Source, using Si-calibrated X-rays of approximate wavelength 0.824 Å and a Mythen 3 position sensitive detector. Samples were diluted in ground silica glass to minimize absorption and then placed in sealed 0.5 mm diameter borosilicate glass capillaries.

Neutron powder diffraction (NPD) data were collected using the POLARIS diffractometer at the ISIS neutron and muon source. Samples were contained in 6 mm diameter vanadium cans sealed under Ar with an indium gasket.

Rietveld refinements of diffraction data was performed using the TOPAS Academic v7 software package.[2] Crystal structures were plotted using VESTA.

X-ray absorption spectroscopy data were collected using the beamline B18 at the Diamond Light Source. The measurements were carried out using the Pt-coated branch of the collimating mirrors with Si(111) monochromator for measurements at the Co and Ru K-edges. Appropriate amounts of sample to achieve an absorption length of 1.6 were mixed with 70 mg of cellulose and pressed into 13 mm diameter pellets. The discs were taped on the sample holder which was subsequently sealed in a plastic bag under argon.

DC magnetization measurements were carried out using a Quantum Design MPMS-3 SQUID device from samples contained in gelatin capsules.

Thermogravimetric analysis (TGA) measurements were performed by heating powder samples at 10°C/min under a 10% H_2/N_2 flow using a Mettler-Toledo MX1 instrument

Iodometric titration was used to determine the average oxidation state of the transition metals within samples. Precisely weighed sample portions (~30 mg) were dissolved in 2:1 HCl:deionized water in presence of excess KI (~300 mg) and the liberated I_2 was titrated against a standardized $Na_2S_2O_3$ solution with a starch indicator being added as the end point approached. A constant argon flow was maintained during the titration to avoid oxidation by air. The reduction of Co and Ru by I_2/I^- (E°=0.54 V) is limited to Co^{2+} and Ru^{3+} respectively and only the sum of a+b in $LaSr_2Co^{2+a}Ru^{3+b}O_xF_y$ could be determined.

3. Characterization of $LaSr_2CoRuO_{5.5}F_{3.5}$

SXRD and NPD data collected from fluorinated $LaSr_2CoRuO_7$ could be indexed with a monoclinic unit cell with reflection conditions consistent with the *A*2/*a* space group (#15) as previously reported for $La_2SrCr_2O_7F_2$.[3] A model was constructed, based on the reported crystal structure of $La_2SrCr_2O_7F_2$, and simultaneously refined against both the SXRD and NPD data. During the refinement, all atomic positions were allowed to refine freely, as were isotropic displacement parameters for all atoms. The NPD data background was fitted by a separate 10-term polynomial function for each detector bank, and a linear absorption correction term. The SXRD data background was fitted using an 8-term polynomial function. The simultaneous refinement of the model to the NPD and SXRD data achieved a good fit as shown in Figures S2 and S3 and described in Table S2. Given that both the X-ray and neutron scattering powers of O and F are very similar, the model was initially refined with only oxygen atoms occupying the anion sites.

Results of the refinement confirm the full occupancy of the tetrahedral interstitial anion site in the rocksalt layer (and full occupancy of all the anion sites) which gives a total of 9 anions per formula unit after fluorination, compared to 7 anions per formula for the starting oxide.

Knowing this, we can determine the anionic composition by measuring the oxidation state of the transition metals and then balancing the total charge of cations by O^{2-} and F^-. Iodometric titrations performed 3 times on sample portions of $LaSr_2CoRuO_7$ and $LaSr_2CoRuO_xF_y$ yielded total transition metal charges of +6.97(4) and +7.48(5), respectively. Those charges are consistent with XANES spectra (**Figure 2**, **Figure S5**) which show that the Ru is in the +5 oxidation state for both compounds, but the Co is in the +2 oxidation state for the all-oxide phase and +2.5 for the fluorinated compound. The combination of those results leads to a charge distribution and anion composition in the fluorinated compound corresponding to $LaSr_2Co^{2.5+}Ru^{5+}O_{5.5}F_{3.5}$.

In addition, the anionic composition was confirmed by a reductive TGA experiment shown in **Figure S6** where the observed mass loss is in line with the expected oxygen loss of 3.75 oxide ions for the following reduction reaction (1), confirmed by X-ray diffraction (Figure S7).

$LaSr_2CoRuO_{5.5}F_{3.5} + 3.75\ H_2 \rightarrow Ru + Co + 0.5\ La_2O_3 + 0.25\ SrO + 1.75\ SrF_2 + 3.75\ H_2O$ **(1)**

Bond valence sums (BVS) were calculated for the 5 crystallographically distinct anion sites in the structure of $LaSr_2CoRuO_{5.5}F_{3.5}$ using parameters for both fluoride and oxide ions, as shown in Table S3. It can be seen that the tetrahedral interstitial interlayer site (F(1)) and the apical anion site (O/F(1)) have the smallest calculated BVS values using either the parameters for fluoride or oxide, consistent with the location of fluoride ions on these sites. We therefore assign the anion distribution in $LaSr_2CoRuO_{5.5}F_{3.5}$ as shown in Table S2, Figure 1 and Figure S4, which is directly analogous to that observed for $La_3Ni_2O_{5.5}F_{3.5}$ and $Sr_3FeRuO_{5.5}F_{3.5}$.[4-5]

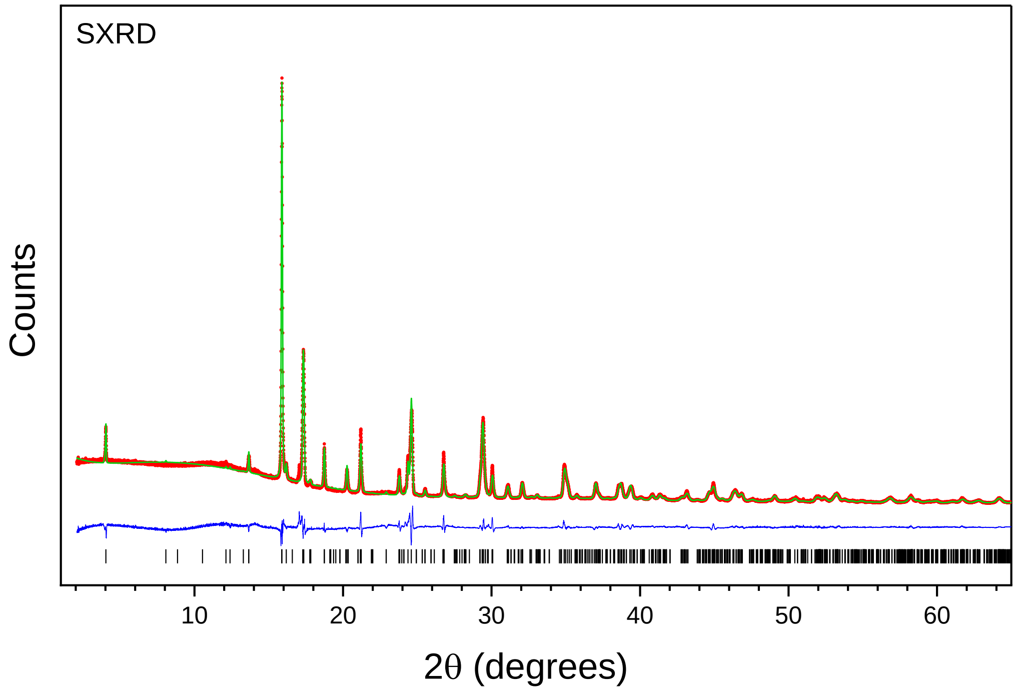

Figure S2. Observed, calculated and difference plots from the structural refinement of an *A*2/*a* symmetry model against SXRD data collected from $LaSr_2CoRuO_{5.5}F_{3.5}$ at room temperature.

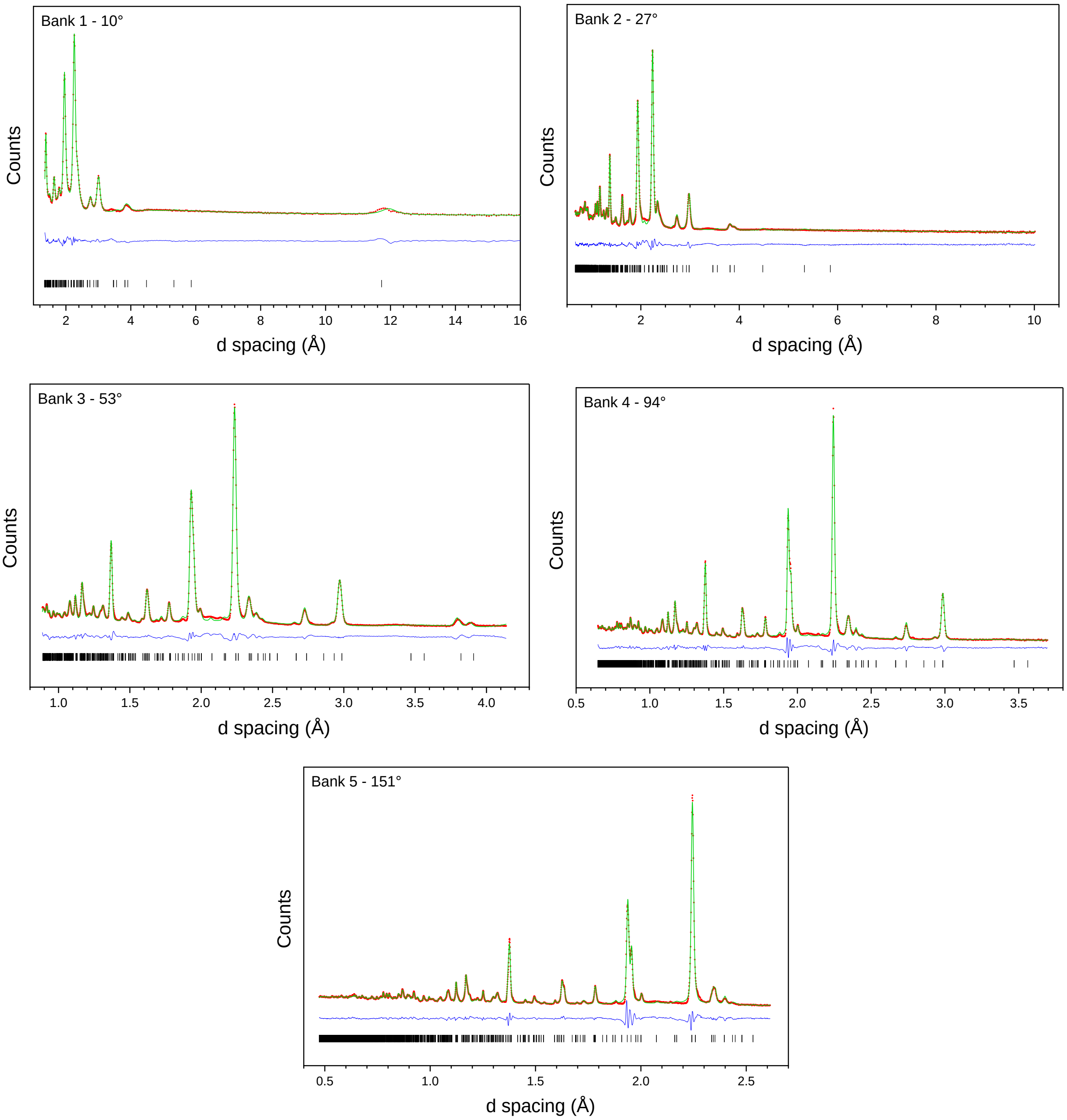

Figure S3. Observed, calculated and difference plots from the structural refinement of an *A*2/*a* symmetry model of $LaSr_2CoRuO_{5.5}F_{3.5}$ against NPD data collected using the 5 detector banks of the POLARIS instrument at room temperature.

Atom	Site	*x*	*y*	*z*	Occupancy	B_{iso} (Å^2)
La/Sr(1)	4*e*	¼	0.754(2)	0	0.333 / 0.666	0.95(5)
La/Sr(2)	8*f*	0.249(10)	0.7566(7)	0.1830(10)	0.333 / 0.666	0.56(3)
Co/Ru	8*f*	0.243(2)	0.2401(10)	0.0852(2)	0.5 / 0.5	0.20(3)
O/F(1)	8*f*	0.265(2)	0.2880(8)	0.1658(10)	0.25 / 0.75	0.61(6)
O(1)	4*e*	¼	0.235(5)	0	1	1.5(2)
O(2)	8*f*	0.956(3)	0.037(3)	0.0930(3)	1	0.4(2)
O(3)	8*f*	0.544(3)	0.461(3)	0.0786(5)	1	0.2(1)
F(2)	8*f*	0.503(2)	0.500(4)	0.2539(7)	1	0.2(1)
$LaSr_2CoRuO_{5.5}F_{3.5}$, space group *A*2/*a* (# 15)<br>a = 5.474(5) Å, b = 5.4845(2) Å, c = 23.437(2) Å, β = 89.97(2) V = 702.52(6) Å^3<br>Formula weight = 628.64 g·mol^{-1}, Z = 4						
Radiation source: Synchrotron X-ray radiation (λ = 0.824 Å), TOF neutron<br>Temperature: 300 K<br>R_{wp} = 5.43%, R_p = 3.95%, R_{exp} = 8.45%						

Table S2. Parameters extracted from the structural refinement of $LaSr_2CoRuO_{5.5}F_{3.5}$ against SXRD and NPD data collected at room temperature.

**Anion site**	**Fluorine BVS**	**Oxygen BVS**	**Site assignment**
Apical O/F(1)	1.23	1.52	Fluorine
Bridging O(1)	1.58	1.95	Oxygen
Equatorial O(2)	1.67	2.08	Oxygen
Equatorial O(3)	1.65	2.03	Oxygen
Interstitial F(2)	1.18	1.29	Fluorine

Table S3. Bond valence sums of the different anion sites in $LaSr_2CoRuO_{5.5}F_{3.5}$ calculated for either a full occupancy by F^- or O^2. Parameters used for the calculations: R_0(O-Sr) = 2.12 Å, R_0(O-La) = 2.15 Å, R_0(O-Co^{2+}) = 1.69 Å, R_0(O-Ru^{5+}) = 1.90 Å, R_0(F-Sr) = 2.02 Å, R_0(F-La) = 2.08 Å, R_0(F-Co^{2+}) = 1.64 Å, R_0(F-Ru^{5+}) = 1.82 Å, b = 0.37 Å.[6]

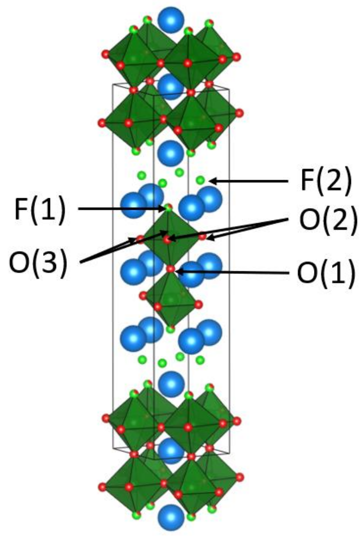

Figure S4. Crystal structure and labelling of anion sites in $LaSr_2CoRuO_{5.5}F_{3.5}$

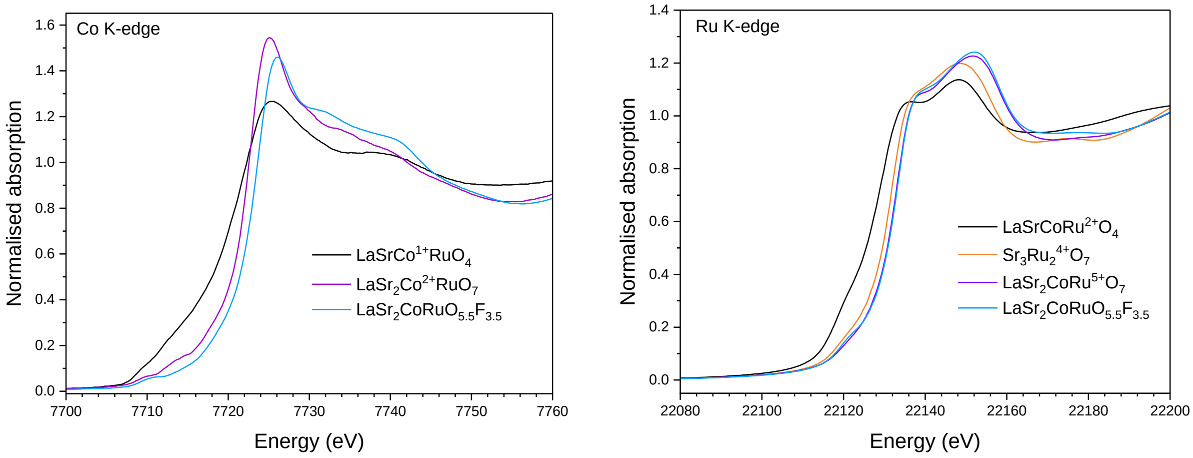

Figure S5. X-ray absorption near edge spectra (XANES) collected at Co and Ru K-edges for $LaSr_2CoRuO_7$ and $LaSr_2CoRuO_{5.5}F_{3.5}$. The spectra are referenced against $LaSr_2Co^{1+}Ru^{2+}O_4$ and $Sr_3Ru_2^{4+}O_7$.

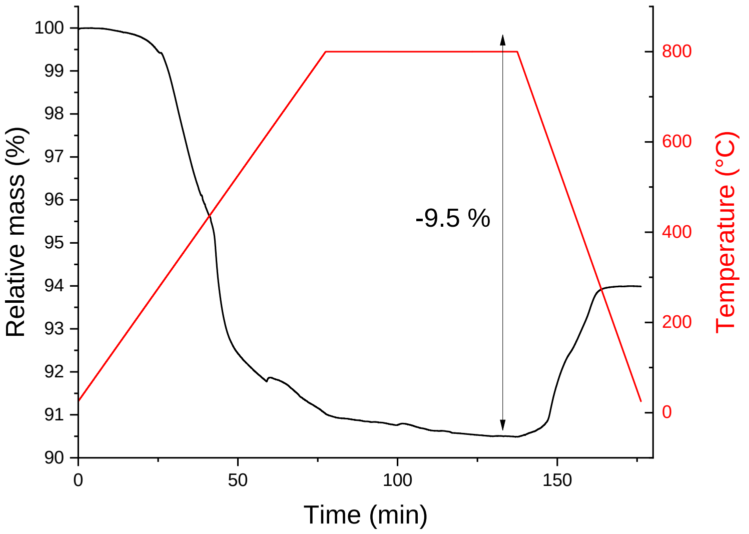

Figure S6. Thermogravimetric analysis data collected from $LaSr_2CoRuO_{5.5}F_{3.5}$ while heated under a flowing 10% H_2, 90% N_2 atmosphere.

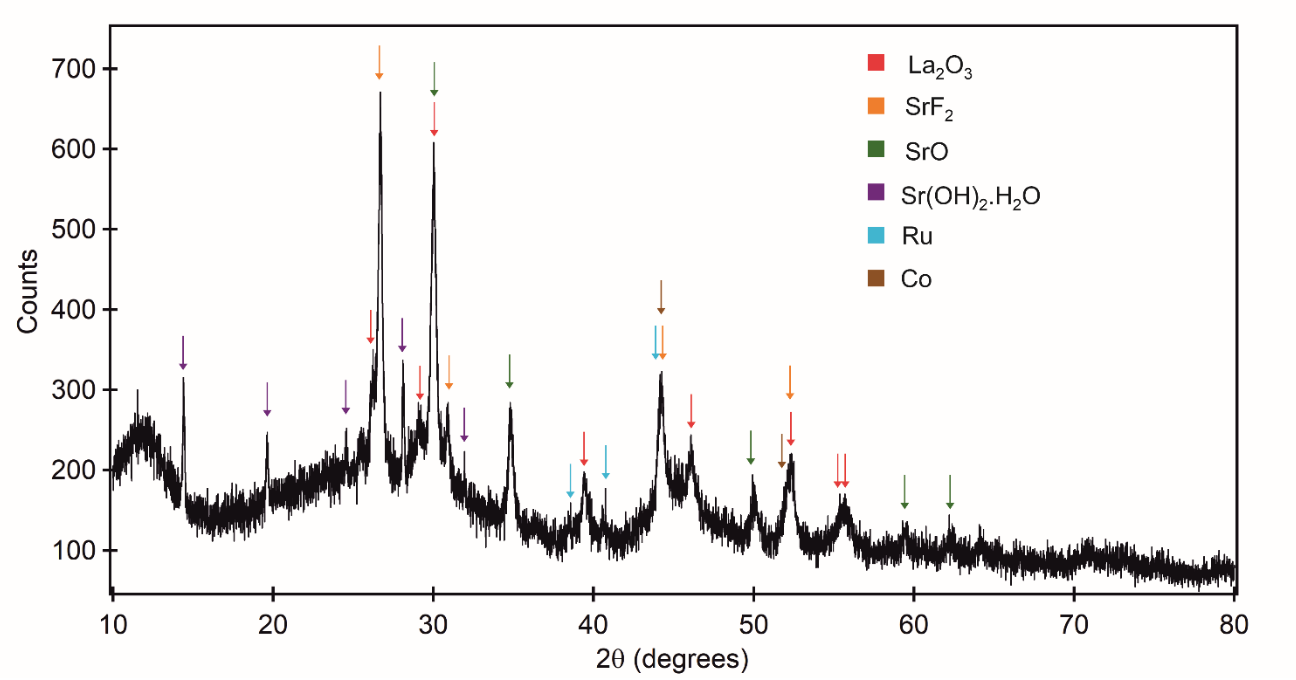

Figure S7. X-ray powder diffraction data collected from the sample after thermogravimetric analysis under a flowing 10% H_2, 90% N_2 atmosphere. Arrows indicate phases consistent with equation (1). The $Sr(OH)_2.H_2O$ observed in the data formed post-reaction, on exposure to air.

4. Characterization of $LaSr_2CoRuO_5F_{1.5}$

SXRD and NPD data collected from the product of reaction between $LaSr_2CoRuO_{5.5}F_{3.5}$ and LiH at 185 °C could be readily indexed using a tetragonal unit cell (a = 4.01 Å, c = 20.13 Å) with extinction conditions consistent with the *I*4/*mmm* space group (#139). A model based on the structure of $LaSr_2CoRuO_7$, but with additional tetrahedral interstitial anion sites in the rock salt interlayer region, was constructed and refined simultaneously against the SXRD and NPD data. During the refinement, all atomic positions were allowed to refine freely, as were isotropic displacement parameters for all atoms. The NPD data background was fitted by a separate 10-term polynomial function for each detector bank, and a linear absorption correction term. The SXRD data background was fitted using an 8-term polynomial function. In the final stages of the refinement, the occupancies of the anion sites were refined, revealing that the interlayer anion site remained fully occupied, while the apical anion site refined to zero occupancy and the bridging anion site refined to half occupancy, corresponding to an overall anion composition of $LaSr_2CoRu(O/F)_{6.5}$. No distinction was made between oxide and fluoride ions during the refinement. An additional series of reflections were observed in neutron diffraction data sets corresponding to the presence of LiF, formed as a by-product during the deintercalation of fluoride ions, so this was added as a second phase to the refinement model.

TGA data collected during the reduction of the sample under a 10% H_2 in N_2 atmosphere (Figure S12) exhibit a mass loss consistent with the loss of 2.25 oxygen atoms consistent with the reaction (2) indicating an overall sample composition of $LaSr_2CoRuO_5F_{1.5}$.

$LaSr_2CoRuO_5F_{1.5} + 2.25\ H_2 \rightarrow Ru + Co + 0.5\ La_2O_3 + 1.25\ SrO + 0.75\ SrF_2 + 2.25\ H_2O$ **(2)**

Co and Ru K-edge XANES spectra (**Figure S11**) reveal an oxidation state of +3 for Ru and an average oxidation state slightly above +1 for Co. When combined with the anion composition determined from the TGA data the overall composition of the phase can be written as $LaSr_2Co^{1.5+}Ru^{3+}O_5F_{1.5}$.

Bond valence sums (BVS) were calculated for the 3 crystallographically distinct, occupied anion sites in the structure of $LaSr_2CoRuO_5F_{1.5}$ using parameters for both fluoride and oxide ions, as shown in Table S5. It can be seen that the tetrahedral interstitial interlayer site (F(1)) has the smallest calculated BVS values using either the parameters for fluoride or oxide, consistent with the location of fluoride ions on this sites. We therefore assign the anion distribution in $LaSr_2CoRuO_5F_{1.5}$ as shown in Table S4, Figure 1 and Figure S10.

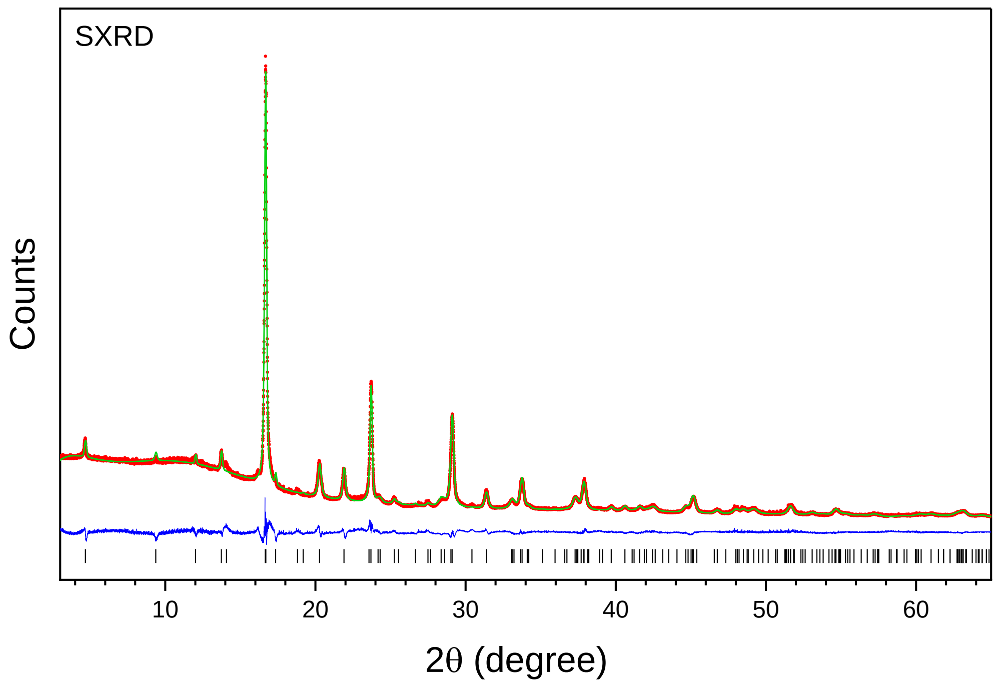

Figure S8. Observed, calculated and difference plots from the structural refinement of an *I4/mmm* symmetry model against SXRD data collected from $LaSr_2CoRuO_5F_{1.5}$ at room temperature. LiF is not visible in the X-ray diffraction data.

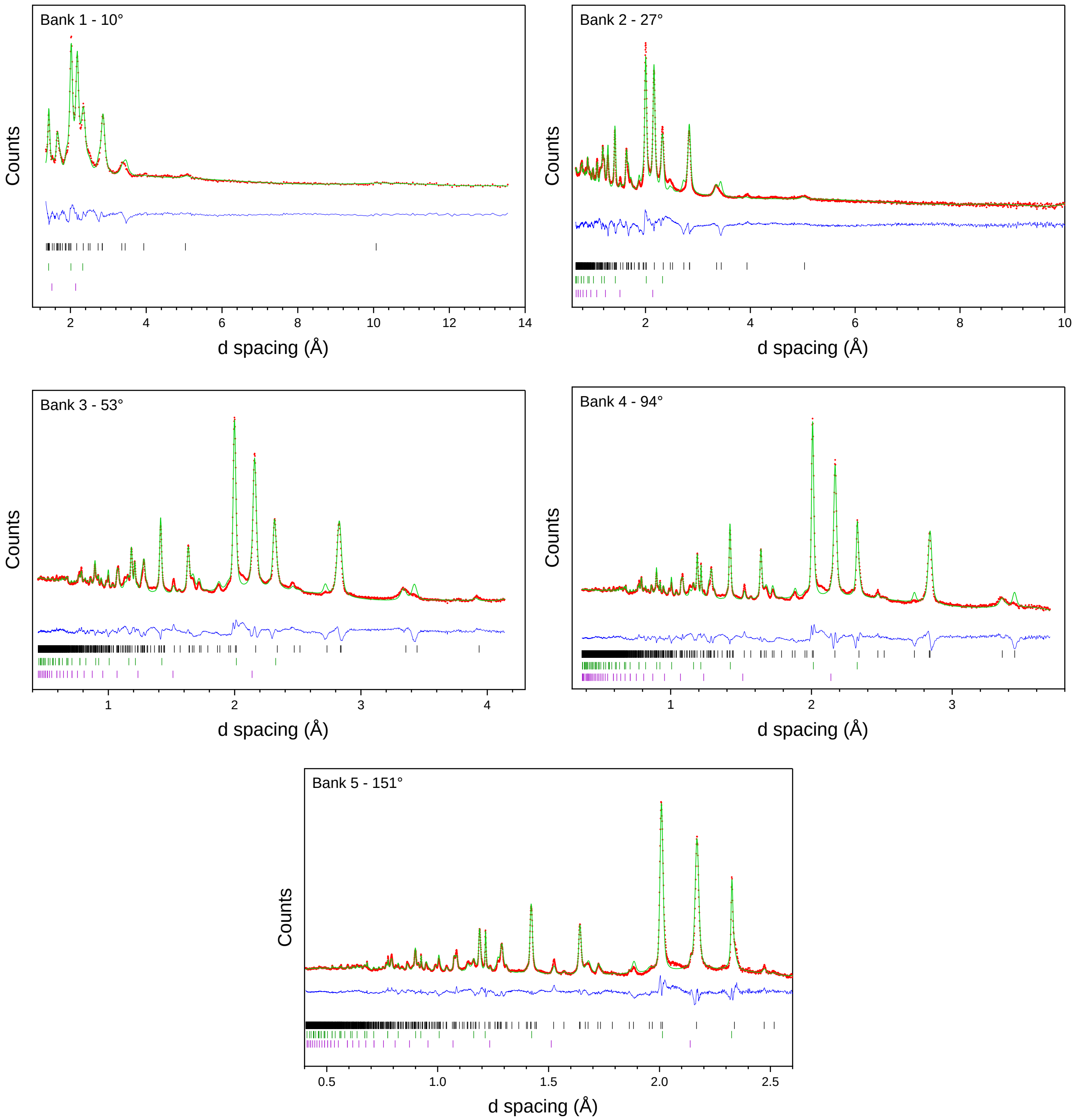

Figure S9. Observed, calculated and difference plots from the structural refinement of an *I4/mmm* symmetry model against NPD data collected from $LaSr_2CoRuO_5F_{1.5}$ using the 5 detector banks of the POLARIS instrument at room temperature. Tick marks correspond to Bragg reflections of the main phase (black), LiF by-product (green) and vanadium sample can (purple).

Atom	site	*x*	*y*	*z*	Occupancy	B_{iso} (Å^2)
La/Sr(1)	2*b*	0	0	½	0.333 / 0.666	0.20(4)
La/Sr(2)	4*e*	0	0	0.3213(2)	0.333 / 0.666	2.0(5)
Co/Ru	4*e*	0	0	0.0918(2)	0.5 / 0.5	1.00(5)
O(1)	8*g*	0	½	0.094(2)	1	1.8(1)
O(2)	2*a*	0	0	0	0.5	2.0(5)
O/F(1)	4*d*	0	½	¼	0.25/0.75	1.9(2)
$LaSr_2CoRuO_5F_{1.5}$, space group *I*4/*mmm* (# 139)<br>*a* = 4.0126(2) Å, *c* = 20.133(2) Å, V = 324.16(3) Å^3<br>Formula weight = 582.65 g·mol^{-1}, Z = 2						
LiF, space group *Fm*-3*m* (#225)<br>*a* = 4.0270(2) Å, V = 65.304(9) Å^3<br>weight percent: 4.6(1)%						
Radiation source: Synchrotron X-ray radiation (λ = 0.824 Å)<br>Temperature: 300 K<br>R_{wp} = 4.39%, R_p = 3.08%, R_{exp}= 7.48%						

Table S4. Parameters extracted from the structural refinement of $LaSr_2CoRuO_5F_{1.5}$ against SXRD and NPD data collected at room temperature.

**Anion site**	**Fluorine BVS**	**Oxygen BVS**	**Site Assignment**
Bridging O(2)	1.51	1.80	Oxygen
Equatorial O(1)	1.64	1.97	Oxygen
Interstitial F(1)	1.25	1.60	Fluorine

Table S5. Bond valence sums of the different anion sites in $LaSr_2CoRuO_5F_{1.5}$ calculated for either a full occupancy by F^- or O^2. Parameters used for the calculations: R_0(O-Sr) = 2.12 Å, R_0(O-La) = 2.15 Å, R_0(O-Co^{2+}) = 1.69 Å, R_0(O-Ru^{4+}) = 1.77 Å, R_0(F-Sr) = 2.02 Å, R_0(F-La) = 2.08 Å, R_0(F-Co^{2+}) = 1.64 Å, R_0(F-Ru^{4+}) = 1.74 Å, *b* = 0.37 Å.[6]

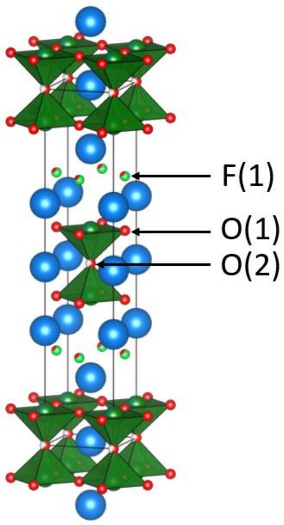

Figure S10. Crystal structure and labelling of anion sites in $LaSr_2CoRuO_5F_{1.5}$.

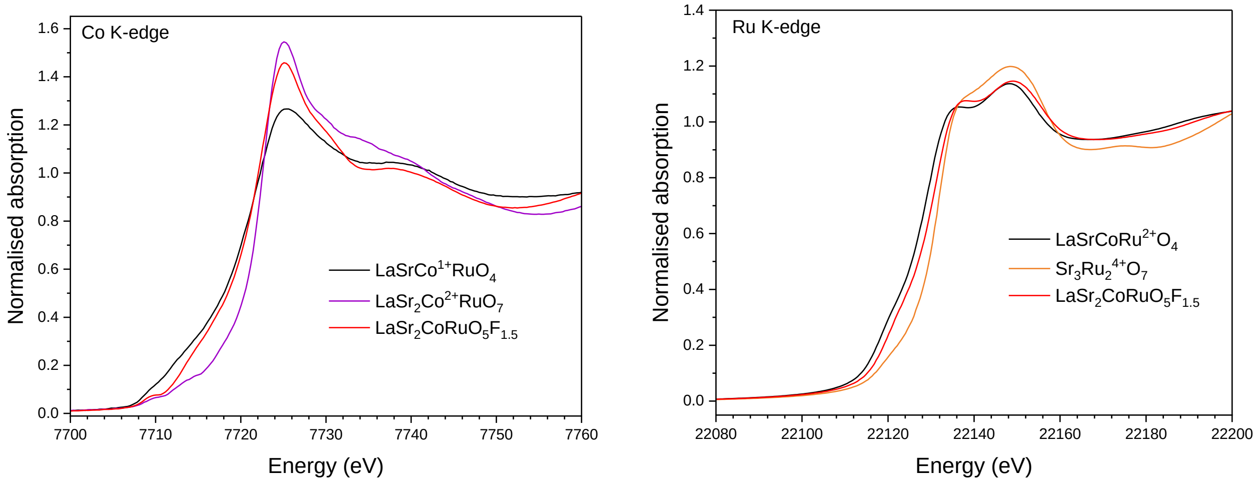

Figure S11. X-ray absorption near edge spectra collected at Co and Ru K-edges from $LaSr_2CoRuO_5F_{1.5}$. The spectra are referenced against $LaSr_2Co^{1+}Ru^{2+}O_4$ $LaSr_2Co^{2+}RuO_7$ and $Sr_3Ru_2^{4+}O_7$.

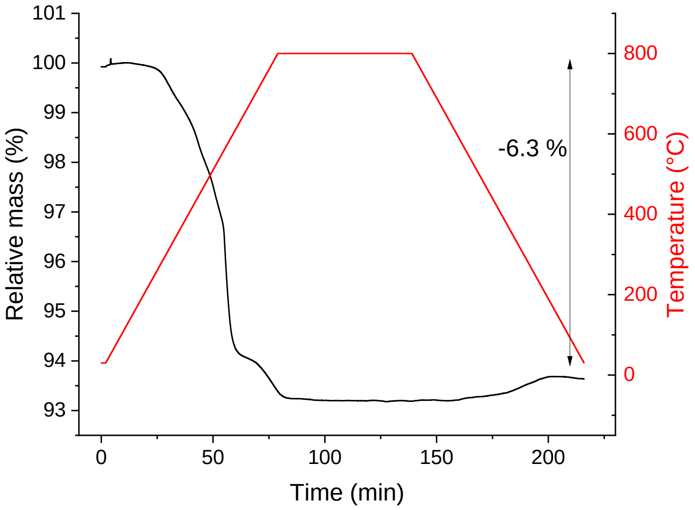

Figure S12. Thermogravimetric analysis curves for $LaSr_2CoRuO_5F_{1.5}$ under flowing 10% H_2/N_2 gas. The sample was reduced to a mixture of Ru, Co, La_2O_3, SrO and SrF_2. The sample was reduced to a mixture of Ru + Co + 0.5 La_2O_3 + 1.25 SrO + 0.75 SrF_2 and the mass loss corresponds to 2.25 oxide ions as expected.

5. Characterization of $LaSr_2CoRuO_{4.5}F_{1.5}$

The SXRD and NPD patterns of the reaction product of $LaSr_2CoRuO_{5.5}F_{3.5}$ with LiH at 230°C could be readily indexed with a tetragonal unit cell (a = 4.02 Å, c = 19.75 Å) and its extinction conditions are consistent with space group *I*4/*mmm* (#139). A model based on the refined structure of $LaSr_2CoRuO_5F_{1.5}$ was constructed and refined simultaneously against the SXRD and NPD data. During the refinement, all atomic positions were allowed to refine freely, as were isotropic displacement parameters for all atoms. The NPD data background was fitted by a separate 10-term polynomial function for each detector bank, and a linear absorption correction term. The SXRD data background was fitted using an 8-term polynomial function. In the final stages of the refinement the occupancies of the anion sites were refined, revealing that the interlayer anion site and equatorial anion site remained fully occupied, while the bridging anion site refined to zero occupancy, yielding a final composition of $LaSr_2CoRu(O/F)_6$. Additional diffraction reflections consistent with the presence of LiF and LaOF were observed in the data, so these were added to the model as secondary phases.

Co and Ru K-edge XANES spectra (**Figure S16**) are consistent with a Co oxidation state of Co^{1+} and a ruthenium oxidation state slightly greater than 2+, which we assign as $Ru^{2.5+.}$ When combined with the TGA data we can formulate the composition of the phase as $LaSr_2Co^{1+}Ru^{2.5+}O_{4.5}F_{1.5}$.

TGA data collected during the reduction of the sample under a 10% H_2 in N_2 atmosphere (**Figure S17**) exhibit a mass loss consistent with the loss of 5.9% corresponding to 2.1 oxygen atoms, which is a little larger than the 4.9% (1.75 Oxygen atoms) expected from equation (3). We attribute this difference to reaction of the sample with moist air while preparing and loading the sample in the apparatus.

$LaSr_2CoRuO_{4.5}F_{1.5} + 1.75\ H_2 \rightarrow Ru + Co + 0.5\ La_2O_3 + 1.25\ SrO + 0.75\ SrF_2 + 1.75\ H_2O$ **(3)**

Bond valence sums (BVS) were calculated for the 2 crystallographically distinct, occupied anion sites in the structure of $LaSr_2CoRuO_{4.5}F_{1.5}$ using parameters for both fluoride and oxide ions, as shown in Table S7. It can be seen that the tetrahedral interstitial interlayer site (F(1)) has the smallest calculated BVS values using either the parameters for fluoride or oxide, consistent with the location of fluoride ions on this site. We therefore assign the anion distribution in $LaSr_2CoRuO_5F_{1.5}$ as shown in Table S6, Figure 1 and Figure S15.

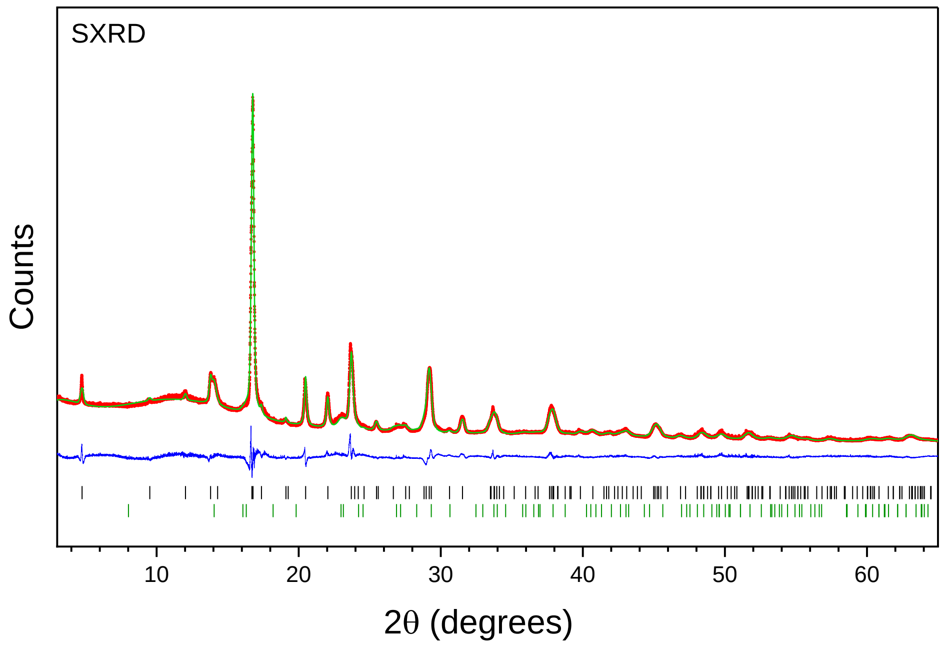

Figure S13. Observed, calculated and difference plots from the structural refinement of an *I4/mmm* symmetry model against SXRD data collected from $LaSr_2CoRuO_{4.5}F_{1.5}$ at room temperature. Tick marks correspond to Bragg reflections of the main phase (black), and LaOF by-product (green). LiF is not visible in the SXRD data.

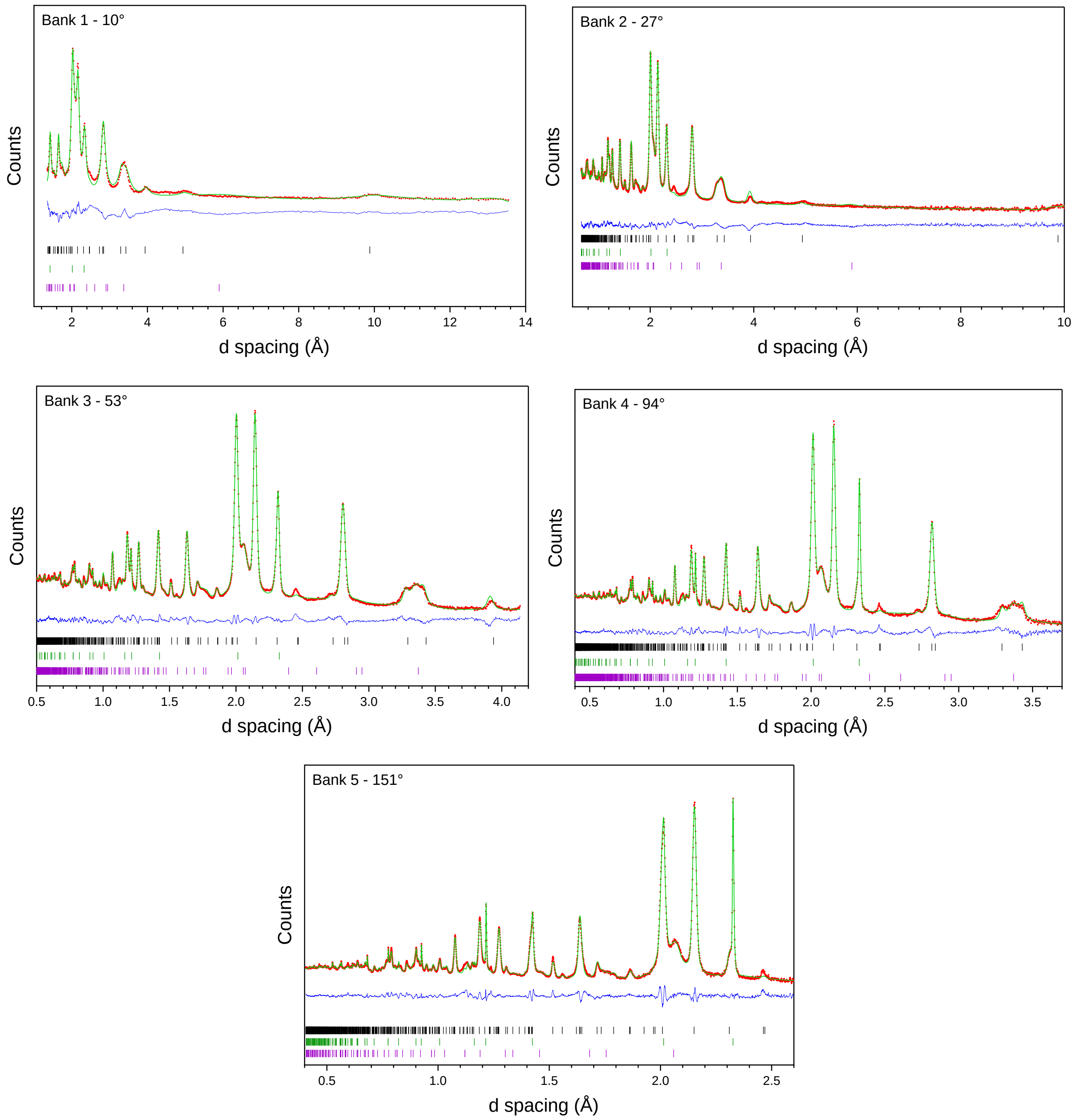

Figure S14. Observed, calculated and difference plots from the structural refinement of an *I4/mmm* symmetry model against NPD data collected from $LaSr_2CoRuO_{4.5}F_{1.5}$ using the 5 detector banks of the POLARIS instrument at room temperature. Tick marks correspond to Bragg reflections of the main phase (black), LiF by-product (green) and LaOF (purple).

Atom	site	*x*	*y*	*z*	Occupancy	B_{iso} (Å^2)
La/Sr(1)	2*b*	0	0	½	0.333 / 0.666	0.20(4)
La/Sr(2)	4*e*	0	0	0.3224(2)	0.333 / 0.666	1.17(4)
Co/Ru	4*e*	0	0	0.0844(2)	0.5 / 0.5	1.47(3)
O(1)	8*g*	0	½	0.0900(2)	1	0.58(2)
F(1)/O	4*d*	0	½	¼	0.75/0.25	2.0(1)
$LaSr_2CoRuO_{4.5}F_{1.5}$, space group *I*4/*mmm* (# 139)<br>*a* = 4.0181(2) Å, *c* = 19.7552970(2) Å, V = 318.98(2) Å^3<br>Formula weight = 574.65 g·mol^{-1}, Z = 2						
LiF, space group *Fm*-3*m* (#225)<br>*a* = 4.0284(2) Å, V = 65.371(5) Å^3<br>weight percent: 4.5(2)%						
LaOF, space group *P*4/*mmm* (#123)<br>*a* = 4.106(2) Å, *c* = 5.910(3) Å<br>weight percent: 22.3(7)%						
Radiation source: Synchrotron X-ray radiation (λ = 0.824 Å)<br>Temperature: 300 K<br>R_{wp} = 3.11%, R_p = 2.51%, R_{exp} = 8.83%						

Table S6. Parameters extracted from the structural refinement of $LaSr_2CoRuO_{4.5}F_{1.5}$ against SXRD and NPD data collected at room temperature.

Anion site	Fluorine BVS	Oxygen BVS	Site assignment
Equatorial O(1)	1.79	2.16	Oxygen
Interstitial F(1)	1.12	1.46	Fluorine

Table S7. Bond valence sums of the different anion sites in $LaSr_2CoRuO_{4.5}F_{1.5}$ calculated for either a full occupancy by F^- or O^{2-}, from which sites could be assigned a specific anion occupancy. Parameters used for the calculations: R_0(O-Sr) = 2.12 Å, R_0(O-La) = 2.15 Å, R_0(O-Co^{2+}) = 1.69 Å, R_0(O-Ru^{4+}) = 1.77 Å, R_0(F-Sr) = 2.02 Å, R_0(F-La) = 2.08 Å, R_0(F-Co^{2+}) = 1.64 Å, R_0(F-Ru^{4+}) = 1.74 Å, *b* = 0.37 Å.[6]

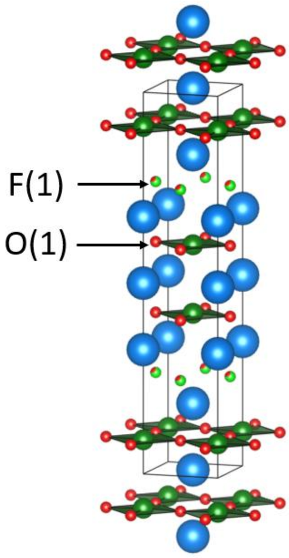

Figure S15. Crystal structure and labelling of anion sites in $LaSr_2CoRuO_{4.5}F_{1.5}$

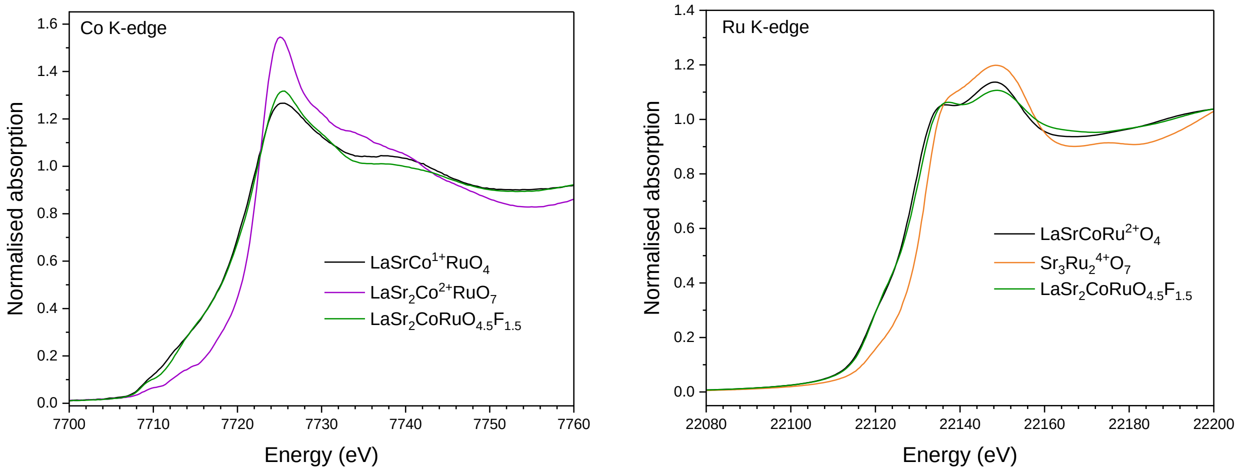

Figure S16. X-ray absorption near edge spectra collected at Co and Ru K-edges for $LaSr_2CoRuO_{4.5}F_{1.5}$. The spectra are referenced against $LaSr_2Co^{1+}Ru^{2+}O_4$ and $Sr_3Ru_2^{4+}O_7$.

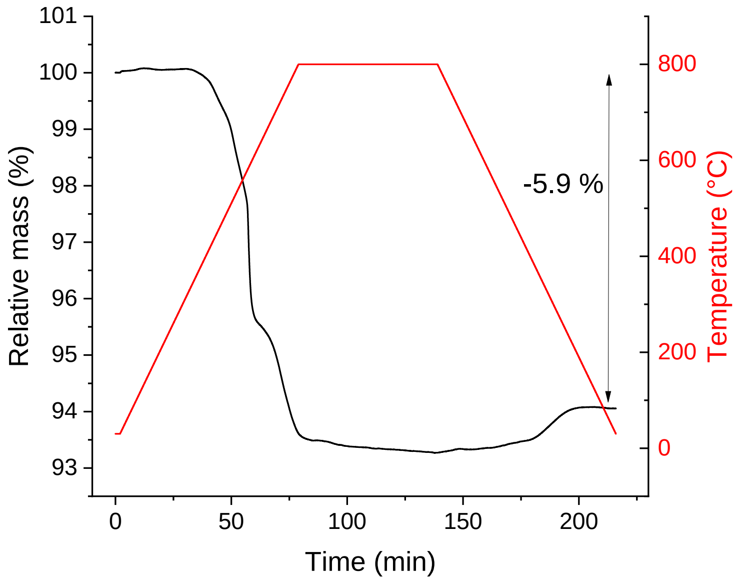

Figure S17. Thermogravimetric analysis curves for $LaSr_2CoRuO_{4.5}F_{1.5}$ under flowing 10% H_2/N_2 gas. The sample was reduced to a mixture of Ru, Co, La_2O_3, SrO and SrF_2. The sample was reduced to a mixture of Ru + Co + 0.5 La_2O_3 + 1.25 SrO + 0.75 SrF_2 and the mass loss corresponds to 2.1 oxide ions whereas a mass loss of 4.9% (1.75 oxide ions) was expected. This small discrepancy might originate from the reaction of the sample with moist air while preparing and loading the sample in the apparatus.

6. Characterization of $LaSr_2CoRuO_{5+\delta}$

SXRD data collected from a sample of $LaSr_2CoRuO_{7-x}$, prepared by reduction with Zr metal at 510 °C, could readily be indexed with an orthorhombic unit cell (*a* = 3.87 Å, *b* = 3.63 Å and *c* = 21.19 Å) with extinction conditions consistent with the *Immm* space group (#71), suggesting a reduced phase analogous to $LaSr_2NiRuO_5$ [1] had been formed.

TGA data collected while heating $LaSr_2CoRuO_{7-x}$ under flowing oxygen (Figure S19) showed a mass gain of 4.76%, corresponding to a gain of 1.66 oxygen atoms. Thus a model was constructed, based on the reported structure of $LaSr_2NiRuO_5$ [1] but with additional oxygen atoms on the O(3) site to yield an overall composition of $LaSr_2CoRuO_{5.3}$. During the refinement, all atomic positions were allowed to refine freely. Isotropic displacement parameters were refined for all atoms, constrained by element. The SXRD data background was fitted using an 8-term polynomial function. This model refined smoothly to give a good fit to the data as shown in Figure S18 and detailed in Table S8

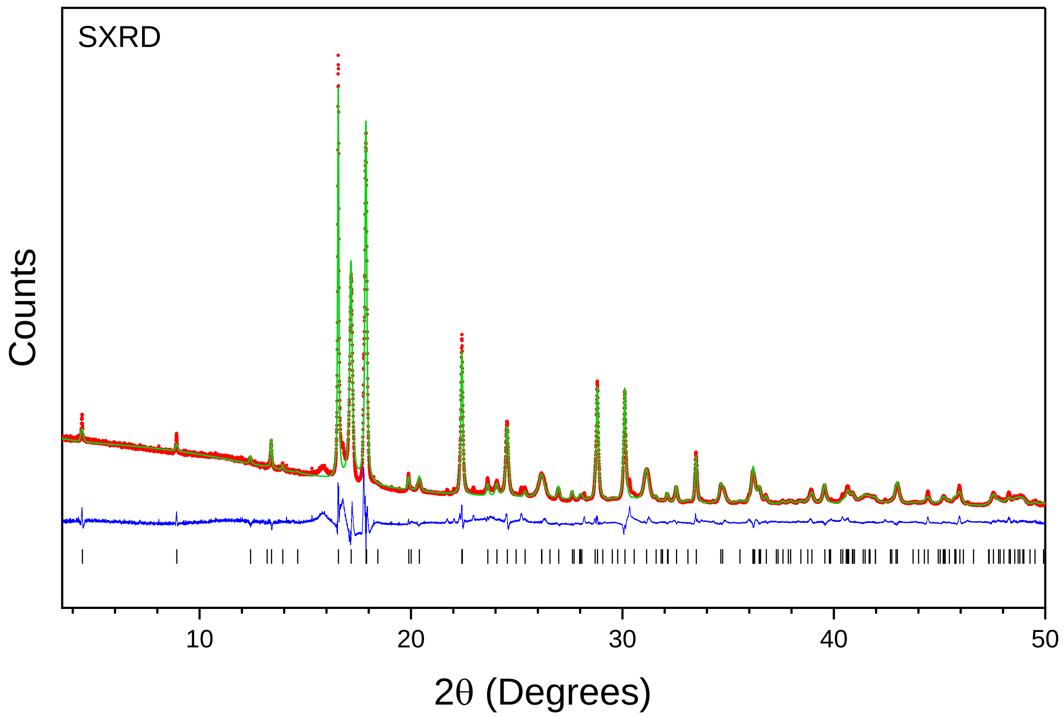

Figure S18. Observed, calculated and difference plots from the structural refinement of an *Immm* symmetry model against SXRD data collected from $LaSr_2CoRuO_{5.3}$ at room temperature.

Atom	site	*x*	*y*	*z*	Occupancy	B_{iso} (Å^2)
La/Sr(1)	2*b*	0	0	½	0.333 / 0.666	0.95(4)
La/Sr(2)	4*i*	0	0	0.3148(2)	0.333 / 0.666	0.95(4)
Co/Ru	4*i*	0	0	0.0977(2)	0.5 / 0.5	0.72(5)
O(1)	2*a*	0	0	0	1	0.83(1)
O(2)	4*j*	½	0	0.0909(5)	1	0.83(1)
O(3)	4*j*	0	½	0.0909(5)	0.15	0.83(1)
O(4)	4*i*	0	0	0.1971(5)	1	0.83(1)
$LaSr_2CoRuO_{5.3}$, space group *Immm* (# 71)<br>a = 3.8743(3) Å, b = 3.6378 (3) Å, c =21.188(2) Å, V = 298.61(4) Å^3<br>Formula weight = 558.95 g·mol^{-1}, Z = 2						
Radiation source: Synchrotron X-ray radiation (λ = 0.824 Å)<br>Temperature: 300 K<br>R_{wp} = 6.21%, R_p = 4.00%, R_{exp} = 2.45%						

Table S8. Crystallographic parameters from structural refinement of $LaSr_2CoRuO_{5.3}$ against SXRD data.

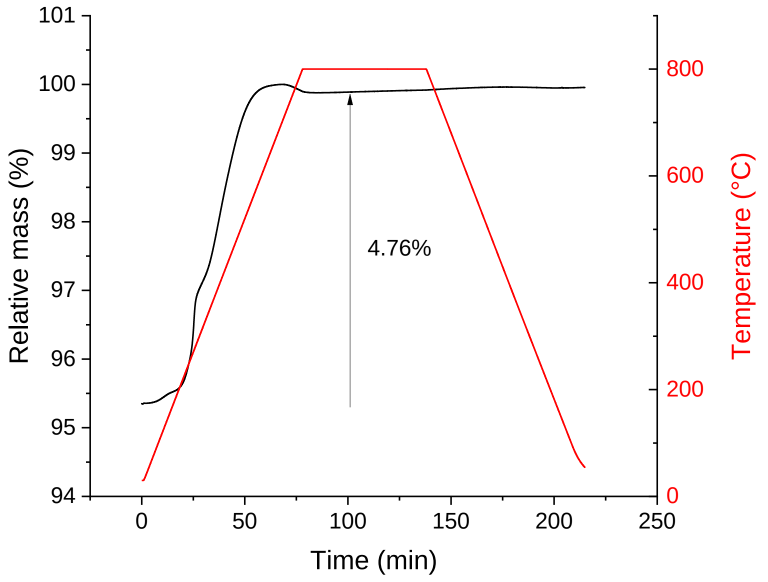

Figure S19. Thermogravimetric analysis curves for $LaSr_2CoRuO_5$ under flowing O_2 gas. The sample was reoxidized back to $LaSr_2CoRuO_7$ with a mass increase corresponding to 1.75 oxide anions whereas 2 were expected.

7. Normalized Co and Ru K-edge XANES spectra

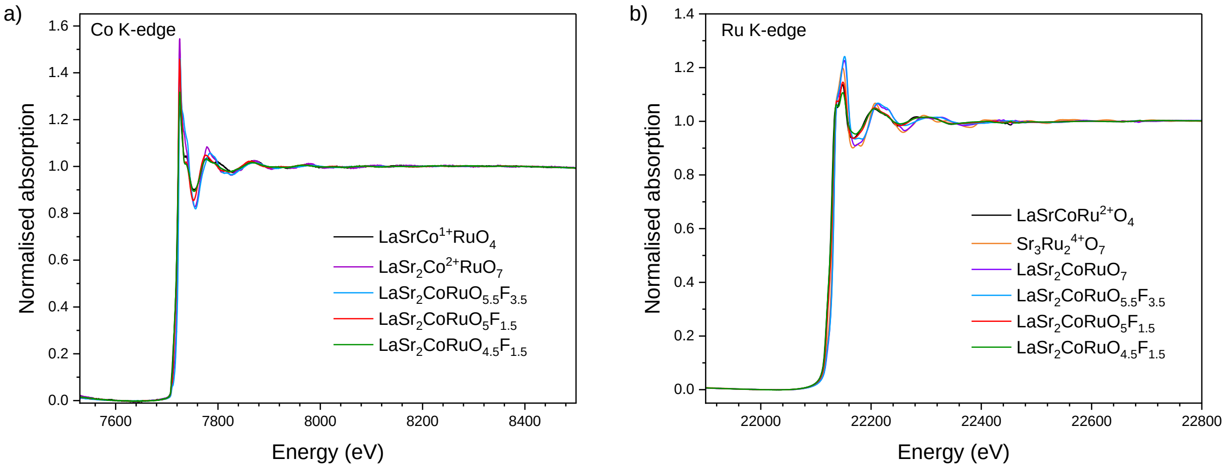

Figure S20. Normalized X-ray absorption spectra at Co and Ru K-edges.

8. Bond Valence Sum Strain (BVSS) calculations for $LaSr_2CoRuO_xF_y$ phases.

As noted in the main text, bond valence sums (BVS) can be used to anticipate which anions in a complex oxide will be removed or exchanged during topochemical reactions.[7] The Bond Valence Sum Strain (BVSS) method is based on the observation that on deintercalation of oxide ions from $A_{n+1}B_nO_{3n+1}$ complex oxides, the redox-active B-cations are reduced, while the redox-inactive A-cations are not. As a consequence, the B-cations need to lower their BVS values by modifying their local coordination spheres so they have longer average bond lengths and/or lower coordination numbers. In contrast the A-cations need to maintain their BVS values, favouring minimal changes to their local coordination spheres. The best compromise between the 'needs' of the A- and B-cations is to remove or exchange anions which make a large contribution to the BVS of the B-cations, while making small contributions to the BVS of the A-cations.

This criterion can be evaluated by calculating the contribution each anion makes to the A-cations (BVS A) and B-cations (BVS B) and then determining the value of (BVS B)-(BVS A), with large positive values of this parameter indicating favorable removal of that particular anion.

If this analysis is performed on $LaSr_2CoRuO_7$ (Table S9) it can be seen that all three anion sites (apical, bridging, equatorial) have total BVS values around two. However, the bond valence sum of the apical anion site has a much larger contribution from the A-cations than the B-cations (BVS B – BVS A = -0.78) indicating that it is unfavorable to remove this anion. This large negative (BVS B) - (BVS A) value for the apical anion site can be attributed to the observation that this anion site is coordinated by 5 A-cations and only 1 B-cation, so it is unsurprising that it contributes more strongly to the BVS of the A-cations in the system. As all apical anion sites in Ruddlesden-Popper phases have A_5B coordinations, we would expect the reductive topochemical deintercalation of the apical anions from such systems to be rare. In contrast both the bridging and equatorial sites have positive (BVS B) - (BVS A) values, with the equatorial site having the largest positive value, indicating the anions on this site will be preferentially deintercalated, if the assumptions BVSS method hold.[7] As noted above, direct reduction of $LaSr_2CoRuO_7$ does deintercalate oxide ions from the equatorial anion sites, in line with this analysis.

$LaSr_2CoRuO_7$	BVS Total	BVS (A)	BVS (B)	BVS (B-A)
Apical	1.72	1.25	0.47	-0.78
Bridging	2.0	0.74	1.26	0.52
Equatorial	2.07	0.7	1.37	0.67

Table S9. Calculated bond valence sums for the three anion sites in $LaSr_2CoRuO_7$ and their separated contributions from the A-cations (Co/Ru) and B-cations (La/Sr) in the structure.

An analogous analysis performed on the fluorinated phase $LaSr_2CoRuO_{5.5}F_{3.5}$ is shown in Table S10. Now it can be seen that the BVS values of the apical and interstitial anions sites are significantly lower than +2, consistent with the presence of fluoride ions on these sites. As the interstitial site has a coordination sphere consisting of only A-cations, this site has a large negative (BVS B) - (BVS A) parameter. In contrast the compression of the (Co/Ru)–O_{ap} bond on fluorination enhances the bonding between the apical anions and the B-cations so that the (BVS B) - (BVS A) parameter is now zero. However, despite this change the bridging and equatorial anion sites have the largest positive values of their (BVS B) - (BVS A)

parameters, indicating the BVSS analysis predicts the removal of anions from equatorial sites on the topochemical reduction of $LaSr_2CoRuO_7$, in contradiction to the observed experimental outcome detailed above.

**$LaSr_2CoRuO_{5.5}F_{3.5}$**	**BVS Total**	**BVS (A)**	**BVS (B)**	**BVS (B-A)**
Apical	1.23	0.62	0.62	0
Bridging	1.94	0.79	1.16	0.37
Equatorial 1	2.05	0.61	1.44	0.84
Equatorial 2	2.03	0.77	1.26	0.49
Interstitial	1.18	1.18	0	-1.18

Table S10. Calculated bond valence sums for the three anion sites in $LaSr_2CoRuO_{5.5}F_{3.5}$ and their separated contributions from the A-cations (Co/Ru) and B-cations (La/Sr) in the structure.

As mentioned in the main text, we attribute the 'failure' of the BVSS method in this instance to strong anion-anion repulsions which are present in $LaSr_2CoRuO_{5.5}F_{3.5}$, but not included within the BVSS analysis method. As also mentioned in the main text the compression of the $B–O_{ap}$ and $O_{ap}–O_{eq}$ bonds on fluorination are signatures of these anion-anion repulsion interactions. A brief survey of a range of topochemically fluorinated n = 2 Ruddlesden-Popper oxides, shown in Table S11, reveals these systems also show analogous bond compressions, suggesting topochemical reduction of these phases is also likely to violate BVSS predictions, indicating the fluorinate-then-reduce sequence of reactions may be used widely to change the regioselectivity of topochemical reduction reactions in Ruddlesden-Popper systems.

	$B–O_{ap}$	$B–O_{br}$	$O_{ap}–O_{eq}$
Sr_3TiRuO_7[4]	2.00	1.98	2.81
$Sr_3TiRuO_7F_2$[4]	1.91	1.99	2.75
$La_2SrCr_2O_7$[8]	2.02	1.98	2.82
$La_2SrCr_2O_7F_2$[3]	1.87	1.92	2.68
$Sr_3Ru_2O_7$[9]	2.01	2.00	2.82
$Sr_3Ru_2O_7F_2$[10]	1.89	2.04	2.67
$La_3Ni_2O_7$[11]	2.22	1.99	2.93
$La_3Ni_2O_{5.5}F_{3.5}$[5]	1.93	1.95	2.67
$LaSr_2CoRuO_7$	2.07	1.98	2.85
$LaSr_2CoRuO_{5.5}F_{3.5}$	1.94	2.00	2.74

Table S11. Calculated anion bond valence sums for a series of $A_3B_2O_7$ n = 2 Ruddlesden-Popper oxides and their fluorinated products.

It is interesting to note that the reduction of $LaSr_2CoRuO_5F_{1.5}$ does follow the BVSS prediction (Table S12) in deintercalating anions from the bridging sites to form $LaSr_2CoRuO_{4.5}F_{1.5}$.

**$LaSr_2CoRuO_5F_{1.5}$**	**BVS Total**	**BVS (A)**	**BVS (B)**	**BVS (B-A)**
Bridging	2.07	0.63	1.44	0.81
Equatorial 1	1.90	0.94	0.96	0.02
Interstitial	1.25	1.25	0	-1.25

Table S12. Calculated bond valence sums for the three anion sites in $LaSr_2CoRuO_5F_{1.5}$ and their separated contributions from the A-cations (Co/Ru) and B-cations (La/Sr) in the structure.

9. Magnetic characterization

Magnetic characterization of $LaSr_2CoRuO_7$

Zero-field cooled and field cooled magnetization data collected from $LaSr_2CoRuO_7$ (Figure S21) can be fit by the Curie-Weiss law (Figure S22) in the range 125 < T/K < 350 K, to yield values C = 5.43(4) cm^3 K mol^{-1}, θ = +52.3(4) K. While the magnetization data fit the mathematical form of the Curie-Weiss law, it is hard to rationalize this very large value of the Curie constant with the Co^{2+} Ru^{5+} oxidation state combination which would be expected to yield a Curie constant of C = 3.75 cm^3 K mol^{-1} according to the spin-only formula. While Co^{2+} centers typically exhibit unquenched orbital contributions to their magnetic moments, for this to account for the observed Curie constant, Co^{2+} would need to exhibit a moment of μ_{eff} = 5.33 μ_B, which is not physically plausible. We therefore attribute the large value of the Curie constant to strong magnetic interactions in the system.

Below 50 K the ZFC and FC data diverge sharply, consistent with a magnetic ordering transition. A magnetization-field isotherm collected at 5 K after cooling from 300 K in an applied field of 50,000 Oe (Figure S23) exhibits hysteresis but does not saturate in an applied field of 50,000 Oe. This suggests that $LaSr_2CoRuO_7$ adopts a canted antiferromagnetic (weak ferromagnetic) or ferrimagnetic state at low temperature.

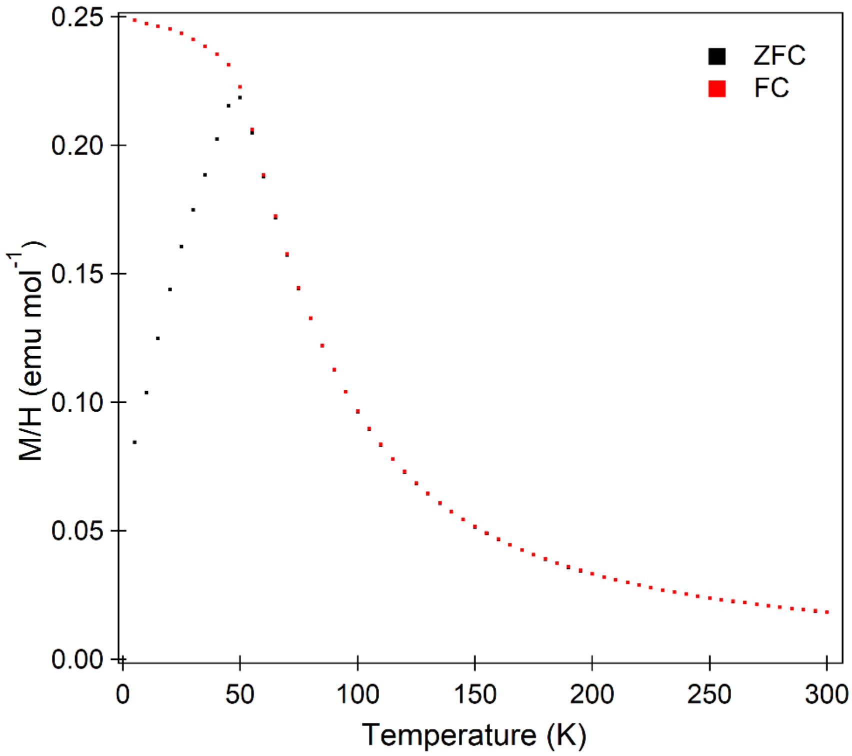

Figure S21. Zero-field cooled and field-cooled magnetization data collected from $LaSr_2CoRuO_7$ as a function of temperature in an applied field of 100 Oe.

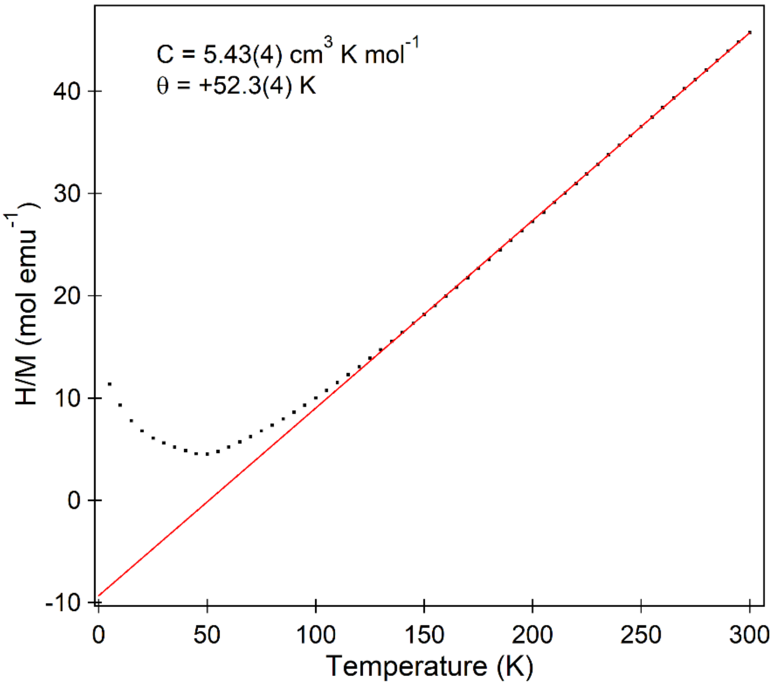

Figure S22. Plot of reciprocal of ZFC magnetization data collected from $LaSr_2CoRuO_7$ as a function of temperature in an applied field of 100 Oe. Fit to data in the temperature range 125 < T/K < 300 yield values of C = 5.43(4) cm^3 K mol^{-1}, θ = +52.3(4) K.

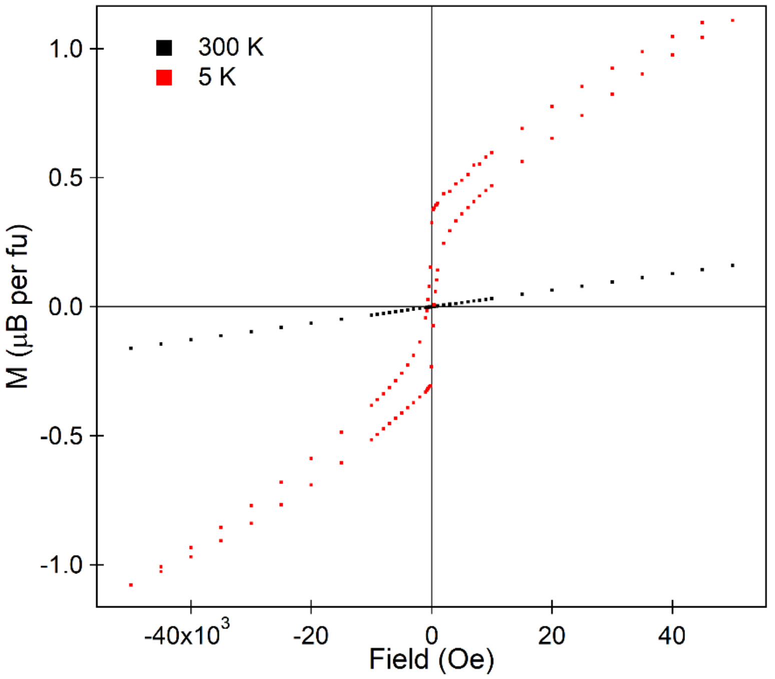

Figure S23. Magnetization data collected from $LaSr_2CoRuO_7$ at 300 K and 5 K, as a function of applied field. The data were collected at 5 K after field cooling from 300 K in an applied field of 50000 Oe.

Magnetic characterization of $LaSr_2CoRuO_{5.5}F_{3.5}$

Zero-field cooled and field cooled magnetization data collected from $LaSr_2CoRuO_{5.5}F_{3.5}$ (Figure S24) can be fit by the Curie-Weiss law (Figure S25) in the range 50 < T/K < 350 K, to yield values C = 1.383(3) cm^3 K mol^{-1}, θ = -38.3(2) K. While the magnetization data fit the mathematical form of the Curie-Weiss law, it is hard to rationalize the value of the Curie constant with the $Co^{2+/3+}$ Ru^{5+} oxidation state combination which would be expected to yield a Curie constant of C = 4.31 cm^3 K mol^{-1} if the Co^{3+} centers are high-spin, or C = 2.81 cm^3 K mol^{-1} if the Co^{3+} centers are low-spin. We attribute the low value of the Curie constant to strong magnetic interactions in the system.

Below 30 K the ZFC and FC data diverge, consistent with a change in magnetic behavior. A magnetization-field isotherm collected at 5 K after cooling from 300 K in an applied field of 50,000 Oe (Figure S26) exhibits hysteresis and are strongly displaced from the origin, suggesting that the magnetic transition observed at 30 K is the freezing of a spin glass. Frustrated glassy magnetic behavior would be consistent with the large degree of disorder (oxide/fluoride disorder, cobalt oxidation state disorder) in the system.

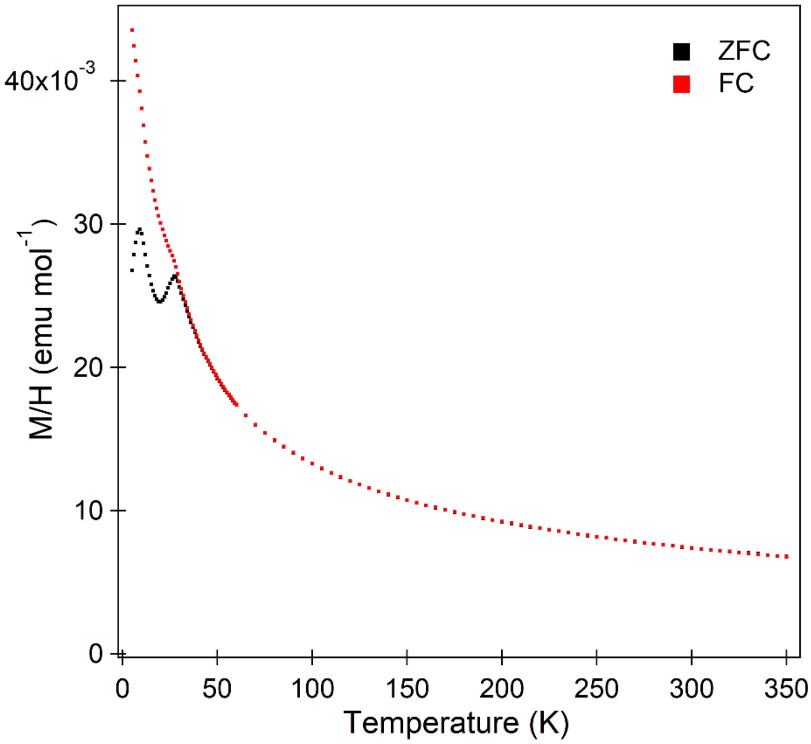

Figure S24. Zero-field cooled and field-cooled magnetization data collected from $LaSr_2CoRuO_{5.5}F_{3.5}$ as a function of temperature in an applied field of 100 Oe.

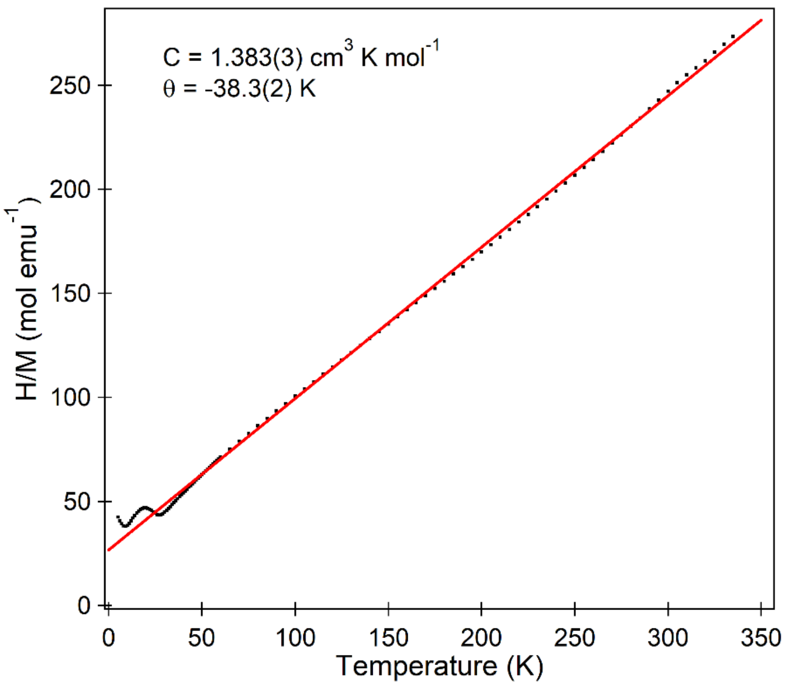

Figure S25. Plot of reciprocal of ZFC magnetization data collected from $LaSr_2CoRuO_{5.5}F_{3.5}$ as a function of temperature in an applied field of 100 Oe. Fit to data in the temperature range 50 < T/K < 350 yield values of C = 1.383(3) cm^3 K mol^{-1}, θ = -38.3(2) K.

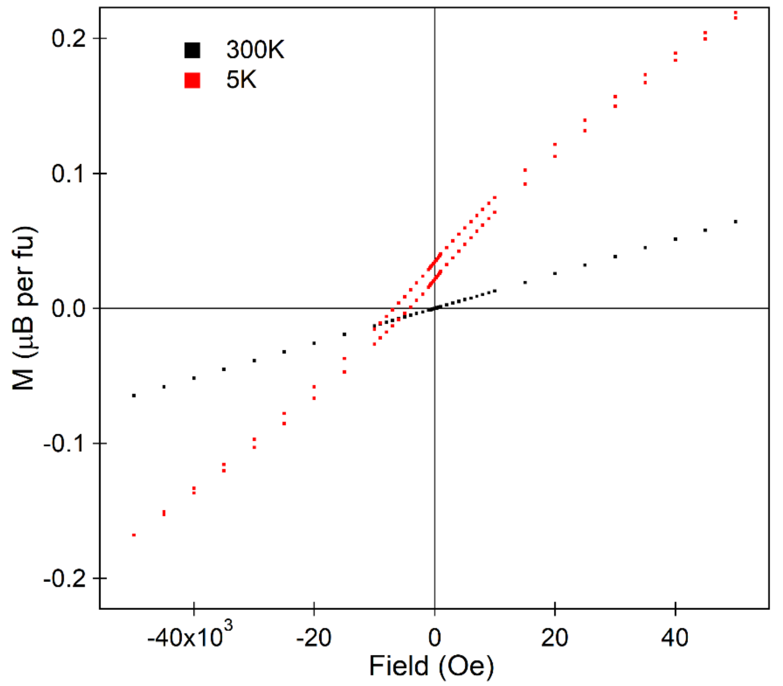

Figure S26. Magnetization data collected from $LaSr_2CoRuO_{5.5}F_{3.5}$ at 300 K and 5 K, as a function of applied field. The data were collected at 5 K after field cooling from 300 K in an applied field of 50000 Oe.

Magnetic characterization of $LaSr_2CoRuO_5F_{1.5}$, $LaSr_2CoRuO_{4.5}F_{1.5}$ and $LaSr_2CoRuO_{5.3}$

Magnetization-field data collected from $LaSr_2CoRuO_5F_{1.5}$, $LaSr_2CoRuO_{4.5}F_{1.5}$ and $LaSr_2CoRuO_{5.3}$ (Figure S27) are sigmoidal, consistent with the presence of small quantities of ferromagnetic, elemental cobalt in samples. Thus, magnetization data were collected from the reduced phases using a ‘ferromagnetic subtraction method’ described in detail in section 10 below.

Figure S28 shows plots of the paramagnetic susceptibility and saturated ferromagnetic moment as a function of temperature for all three reduced phases. It can be seen that the data from all three samples follows the same general form. At high temperatures ‘paramagnetic’ behavior is observed with the paramagnetic susceptibility of all three samples being fit by the Curie-Weiss law, as shown in Figure S29. The Curie constants extracted from these fits are much greater than those expected from the spin-only moments of the Co and Ru centers, again suggesting there are strong magnetic interactions in these systems over the whole temperature range measured.

It is interesting to note that the Curie constants extracted for $LaSr_2CoRuO_{4.5}F_{1.5}$ and $LaSr_2CoRuO_{5.3}$ are similar (4.73 and 4.53 cm^3 K mol^{-1} respectively) consistent with their similar transition-metal oxidation states. However, $LaSr_2CoRuO_{5.3}$ exhibits a larger Weiss constant (θ = -147.3 K) and deviates from the Curie-Weiss law at high temperature (T_{dev} ~ 175 K) than $LaSr_2CoRuO_{4.5}F_{3.5}$ (θ = -48.3 K; T_{dev} ~ 95 K) suggesting that the connectivity of the $(Co/Ru)O_4$ units – 1D in $LaSr_2CoRuO_{5.3}$, 2D in $LaSr_2CoRuO_{4.5}F_{1.5}$ – strongly influence the strength of the magnetic couplings in these systems.

At low temperature the paramagnetic susceptibility of all three samples plateaus and there is an associated sharp increase in the saturated ferromagnetic moments of the materials, indicating a change in magnetic behavior. Magnetization-field data collected from the reduced samples after cooling from 300 K in an applied field of 50,000 Oe, exhibit hysteresis and are displaced from the origin indicating a significant glassy component to the low-temperature magnetic states of all three reduced phases.

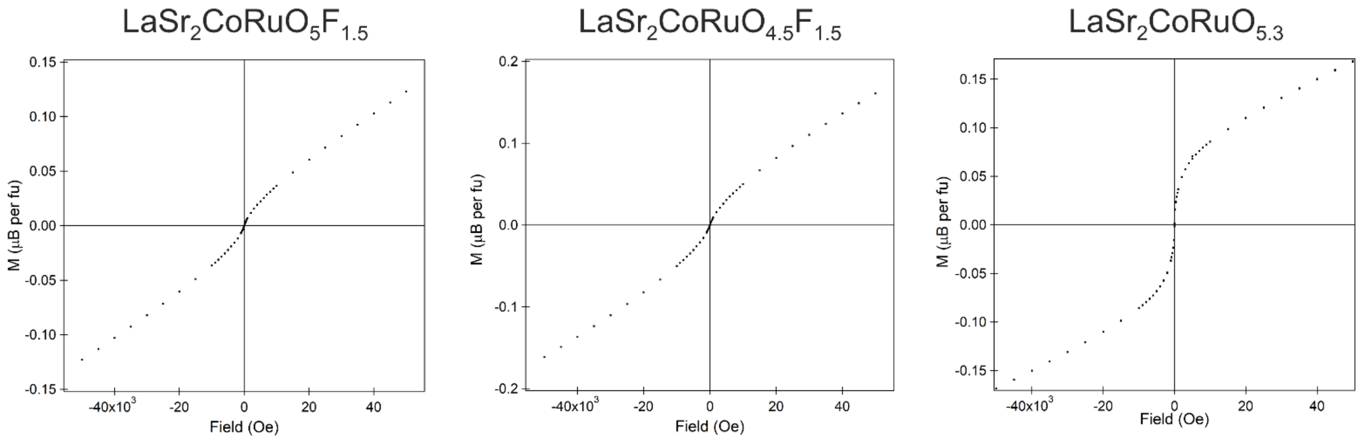

Figure S27. Magnetization-field data collected at 300 K from $LaSr_2CoRuO_5F_{1.5}$, $LaSr_2CoRuO_{4.5}F_{1.5}$ and $LaSr_2CoRuO_{5.3}$ exhibit sigmoidal behavior, consistent with the presence of a ferromagnetic elemental cobalt impurity.

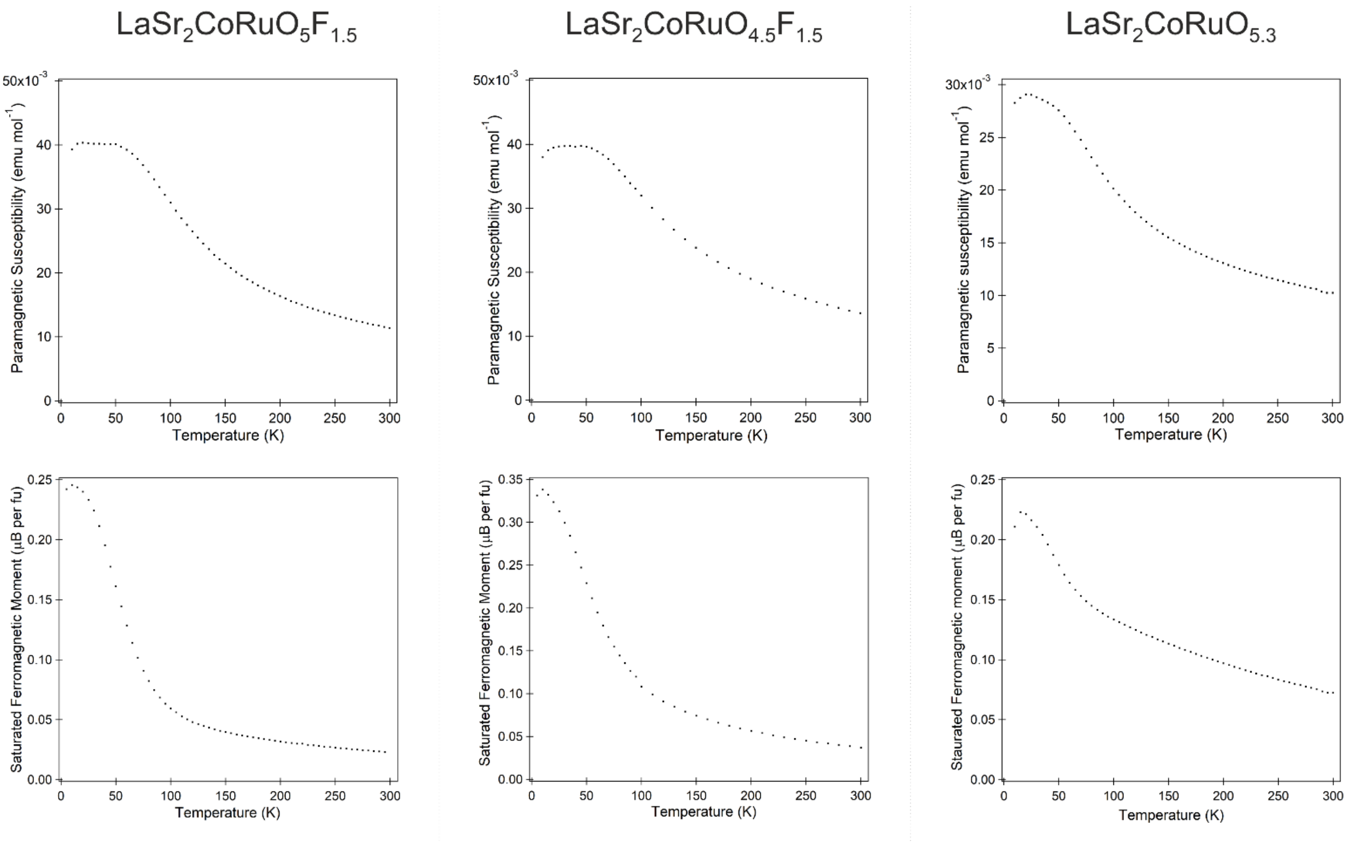

Figure S28. Plots of paramagnetic susceptibility and saturated ferromagnetic moment, as a function of temperature, for $LaSr_2CoRuO_5F_{1.5}$, $LaSr_2CoRuO_{4.5}F_{1.5}$ and $LaSr_2CoRuO_{5.3}$ as determined by the ‘ferrosubtraction method’ described in section 10 below.

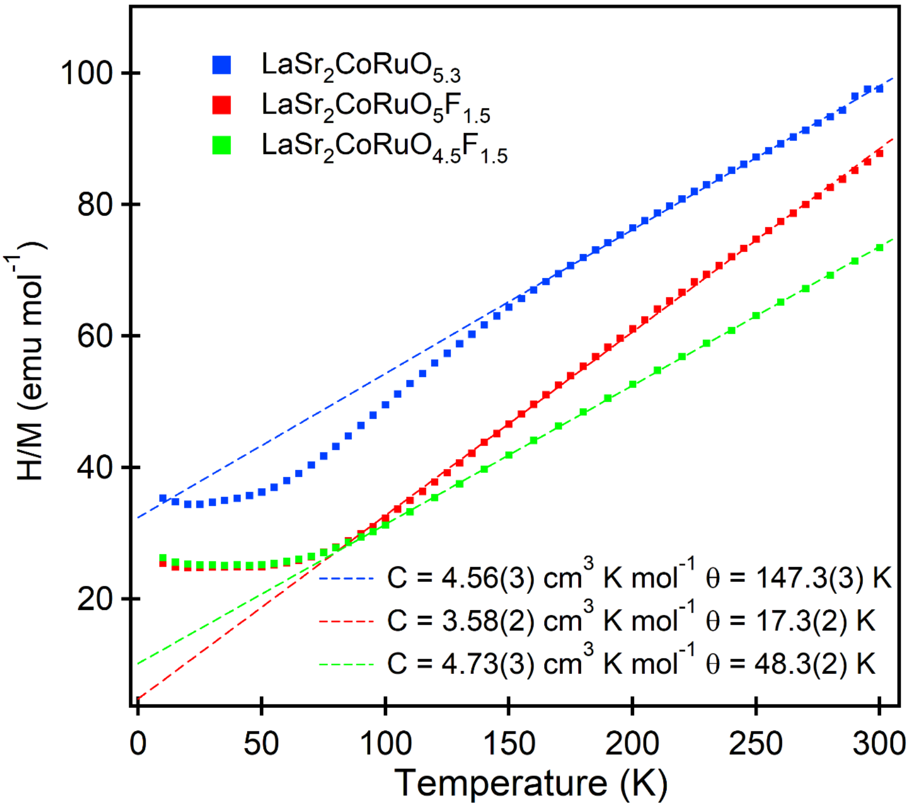

Figure S29. Plots of the inverse paramagnetic susceptibilities of $LaSr_2CoRuO_5F_{1.5}$, $LaSr_2CoRuO_{4.5}F_{1.5}$ and $LaSr_2CoRuO_{5.3}$ against temperature. Data from $LaSr_2CoRuO_{5.3}$ can be fit by the Curie-Weiss law in the range $175 < T/K < 300$ to yield C = 4.56(3) cm^3 K mol^{-1}, θ = -147.3(3) K; $LaSr_2CoRuO_5F_{1.5}$ fit in the range $90 < T/K < 300$ to yield C = 3.58(2) cm^3 K mol^{-1}, θ = -17.3(2) K; $LaSr_2CoRuO_{4.5}F_{1.5}$ fit in the range $95 < T/K < 300$ to yield C = 4.73(3) cm^3 K mol^{-1}, θ = -48.3(2) K

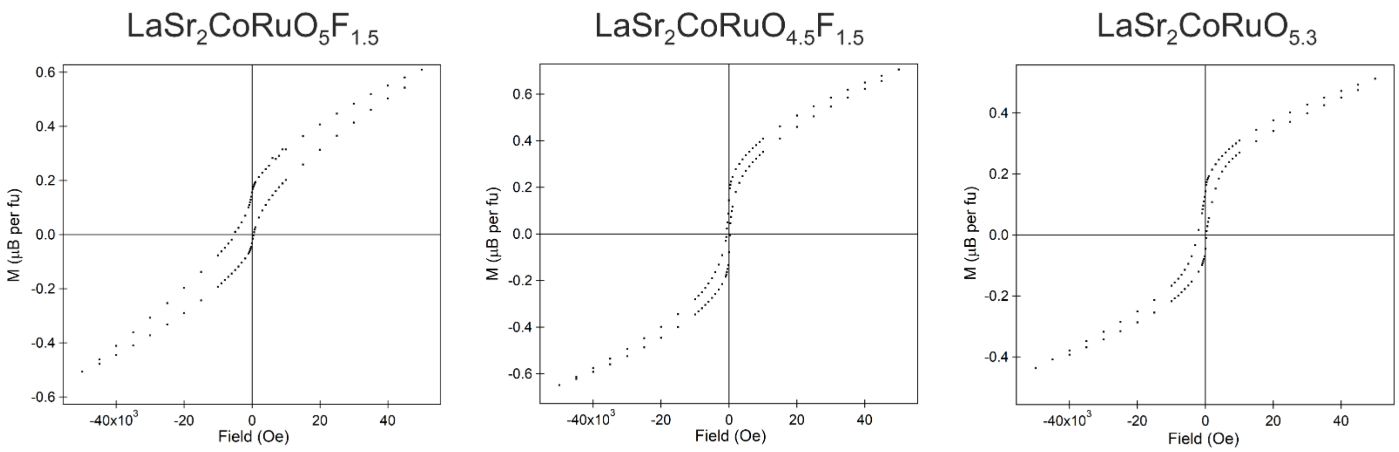

Figure S30. Magnetization-field data collected at 5 K after cooling in 50000 Oe from 300 K, from $LaSr_2CoRuO_5F_{1.5}$, $LaSr_2CoRuO_{4.5}F_{1.5}$ and $LaSr_2CoRuO_{5.3}$.

10. Magnetic measurements in the presence of elemental Co impurities via the 'ferrosubtraction' method

Procedure used to measure the magnetization of samples containing elemental cobalt:
The magnetization of elemental Co is observed to saturate in applied magnetic fields of more than 2 T. Thus, the paramagnetic susceptibility of a bulk sample can be measured in the presence of elemental Co impurities by measuring the gradient of magnetization-field isotherms in applied fields larger than 2 T. As shown in Figure S31.

To this end the magnetization of samples was measured in a series of 5 fields between 3 T and 5 T. The magnetization vs. field data were fitted to a linear function, the gradient of which is the paramagnetic susceptibility of the bulk sample and the intercept is the saturated ferromagnetic moment of the sample. Data points with large errors were excluded from fits. All fits had at least 4 data points. This procedure was repeated at 5 K intervals between 5 K and 300 K to measure the temperature dependent susceptibility of samples.

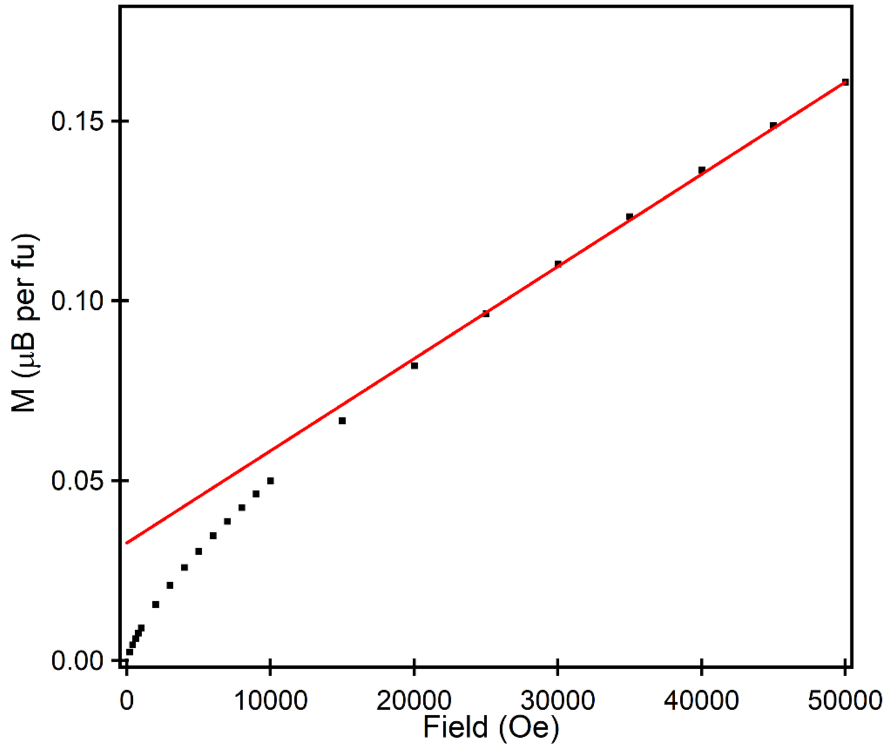

Figure S31. Magnetization of $LaSr_2CoRuO_{4.5}F_{1.5}$ measured as a function of applied field at 300 K. A linear fit to high-field region (H > 25000 Oe) yields a gradient which is the paramagnetic susceptibility of the sample, and an intercept which is the saturated ferromagnetic moment of the sample.

11. References

[1] Z. Y. Xu, L. Jin, J. K. Backhaus, F. Green, M. A. Hayward, *Inorg. Chem.* **2021**, *60*, 14904–14912.

[2] A. A. Coelho, *J. Appl. Crystallogr.* **2018**, *51*, 210–218.

[3] R. Zhang, G. Read, F. Lang, T. Lancaster, S. J. Blundell, M. A. Hayward, *Inorg. Chem.* **2016**, *55*, 3169–3174.

[4] F. Denis Romero, P. A. Bingham, S. D. Forder, M. A. Hayward, *Inorg. Chem.* **2013**, *52*, 3388–3398.

[5] R. Zhang, M. S. Senn, M. A. Hayward, *Chem. Mater.* **2016**, *28*, 8399–8406.

[6] I. D. Brown, D. Altermatt, *Acta Crystallogr., Sect. B : Strucut. Sci.* **1985**, *B41*, 244–247.

[7] M. A. Hayward, *Semiconductor Science and Technology* **2014**, *29*, 064010.

[8] R. Zhang, B. M. Abbett, G. Read, F. Lang, T. Lancaster, T. T. Tran, P. S. Halasyamani, S. J. Blundell, N. A. Benedek, M. A. Hayward, *Inorg. Chem.* **2016**, *55*, 8951–8960.

[9] Q. Huang, J. W. Lynn, R. W. Erwin, J. Jarupatrakorn, R. J. Cava, *Phys. Rev. B* **1998**, *58*, 8515–8521.

[10] R. K. Li, C. Greaves, *Phys. Rev. B* **2000**, *62*, 3811–3815.

[11] C. D. Ling, D. N. Argyriou, G. Q. Wu, J. J. Neumeier, *J. Solid State Chem.* **2000**, *152*, 517–525.
